# The impact of obesity-related systemic inflammation on the efficacy, toxicity, and biomarkers of immune checkpoint inhibitors in lung cancer: from mechanisms to clinical management

**DOI:** 10.3389/fimmu.2026.1757711

**Published:** 2026-02-03

**Authors:** Yewei Cai, Tianxing Ni

**Affiliations:** Department of Cardiothoracic Vascular Surgery, The First People’s Hospital of Xiaoshan District, Hangzhou, Zhejiang, China

**Keywords:** biomarkers, body composition, immune checkpoint inhibitors, immunotherapy, nflammation, lung cancer, obesity, tumor microenvironment

## Abstract

Immune checkpoint inhibitors (ICIs) have revolutionized the treatment landscape of lung cancer, yet the heterogeneity in their efficacy and toxicity among different patients remains a significant clinical challenge. Obesity, as a global epidemic associated with chronic low-grade systemic inflammation and complex immunometabolic disturbances, has been identified as a crucial regulatory factor in cancer immunotherapy response. This review aims to systematically and deeply explore the intricate network of interactions between obesity, lung cancer, and immunotherapy. We not only examine the molecular and cellular mechanisms by which obesity-related inflammation influences ICI efficacy through remodeling the tumor microenvironment, altering systemic immune status, and modulating the gut microbiota, but also comprehensively assess its complex impact on clinical outcomes of ICI (including the controversial “obesity paradox” phenomenon) and immune-related adverse events (irAEs), particularly those uniquely associated with endocrine toxicity. Simultaneously, we systematically review novel biomarkers centered around obesity-related inflammatory parameters and body composition (such as circulating adipokines and radiomic features) and their application in integrative predictive models. Finally, based on available evidence, we propose multidisciplinary, longitudinal clinical management strategies tailored for obese lung cancer patients and envision novel combination treatment directions targeting the obesity-inflammation axis, aiming to provide theoretical support and practical guidance for achieving more precise, individualized immunotherapy.

## 1 Introduction

Lung cancer is the leading cause of cancer-related deaths globally, posing a severe prevention and treatment challenge. Despite the landmark advances in recent years achieved by immune checkpoint inhibitors (ICIs), particularly drugs targeting the PD-1/PD-L1 and CTLA-4 pathways in the treatment of advanced non-small cell lung cancer (NSCLC) and some small cell lung cancer (SCLC) (1), significantly extending the survival of some patients, a sobering reality remains: there is immense interindividual variability (2) in patient treatment responses. Currently, aside from limited indicators like PD-L1 tumor proportion score (TPS) (3) and tumor mutation burden (TMB) (4), clinical practice still lacks sufficiently reliable and comprehensive predictive biomarkers to accurately identify potential beneficiaries, leading to a substantial number of patients possibly bearing unnecessary economic burdens and medication toxicities.

Simultaneously, another global public health issue—obesity, with its prevalence rising continuously, has emerged as a significant metabolic disorder affecting the occurrence, progression, and treatment response of various cancers, including lung cancer (5). In the past, obesity was simply viewed as a storage state of energy surplus; however, it is now widely recognized within academia as a persistent chronic low-grade systemic inflammatory state. Adipose tissue, especially dysfunctional visceral adipose tissue, is a highly active endocrine and immune organ, secreting abundant adipokines (like leptin and adiponectin) (6), inflammatory cytokines (such as TNF-α and IL-6) (7), and chemokines, systematically altering the host’s immune homeostasis and metabolic balance. This obesity-driven immunometabolism disorder profoundly and complexly interacts with the efficacy and toxicity of lung cancer immunotherapy (8).

Particularly intriguing is the resurfacing focus on the “obesity paradox” (9) in the era of immunotherapy—a phenomenon where, despite being a clear risk factor for more than ten types of cancer, retrospective studies (10–12) found that high body mass index (BMI) might correlate with better survival benefits in lung cancer patients receiving ICI treatment. This seemingly contradictory phenomenon challenges conventional understanding of the relationship between obesity and cancer outcomes, indicating potential unique biological mechanisms at play. Therefore, systematically elucidating how obesity, through inflammation as a core bridge, affects the entire process of lung cancer immunotherapy holds immense theoretical and clinical significance.

This review aims to systematically explore the profound influences of obesity-related systemic inflammation on the efficacy, toxicity, and biomarkers of lung cancer ICIs from a multi-dimensional and integrative perspective. By integrating recent breakthroughs in basic research and findings in clinical practice, we investigate the potential molecular and immunological mechanisms underlying these interactions, evaluate the complex relationship between obesity and therapeutic efficacy and toxicity of immunotherapy (especially endocrine immune-related adverse events), comprehensively summarize emerging biomarkers and predictive models based on obesity-related parameters, and ultimately aim to construct precise, longitudinal clinical management strategies for obese lung cancer patients. This comprehensive analysis aims to provide new perspectives and ideas to overcome the heterogeneity of therapeutic efficacy and realize true individualized treatment in the era of lung cancer immunotherapy.

## 2 Obesity, inflammation, and tumor microenvironment in lung cancer

### 2.1 Pathophysiological basis of obesity-related inflammation

**Figure 1:**
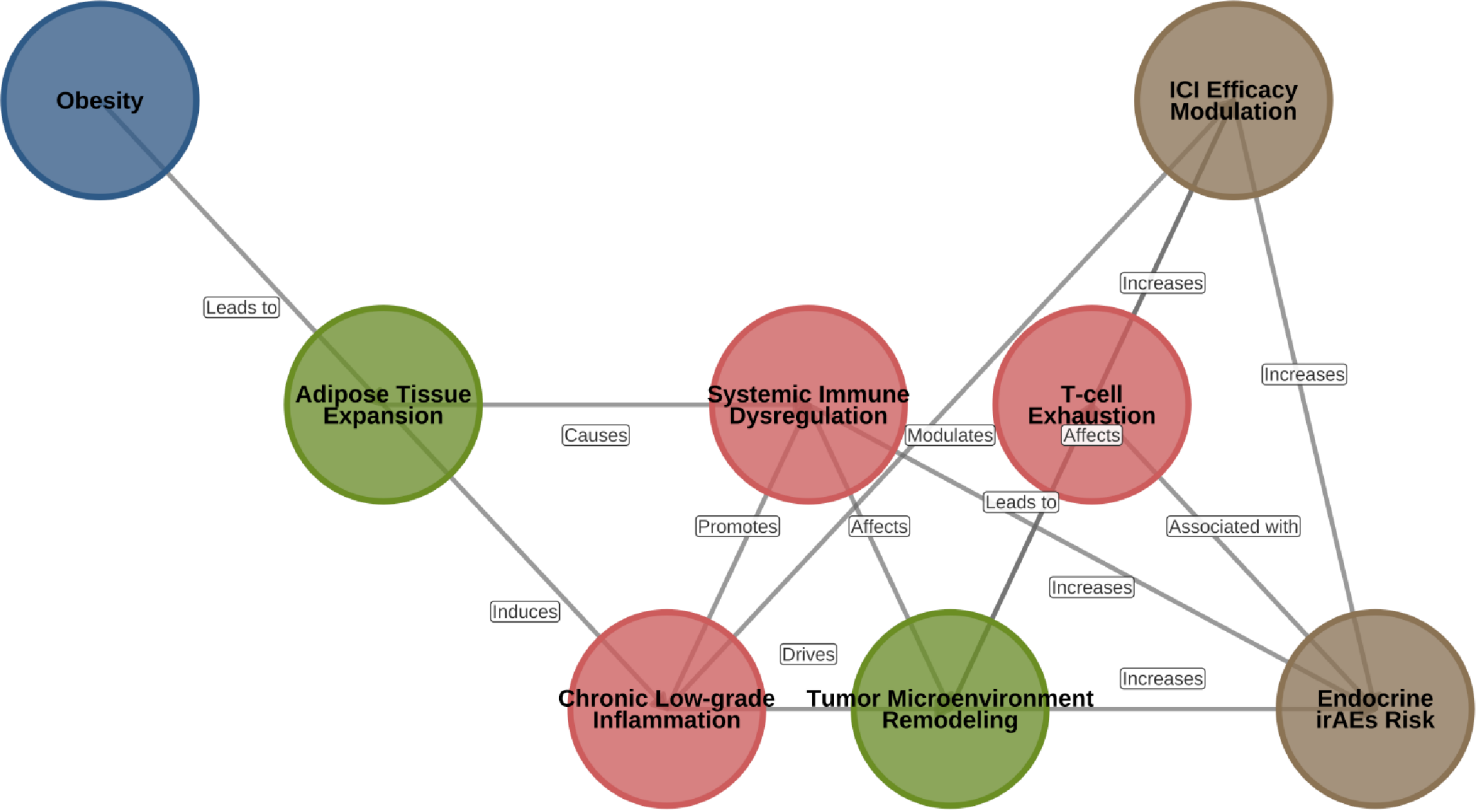
The obesity-inflammation-immunotherapy axis in lung cancer. This schematic diagram illustrates the conceptual framework linking obesity, systemic inflammation, and lung cancer immunotherapy. The network depicts: (1) Expansion of dysfunctional adipose tissue leading to chronic low-grade inflammation characterized by adipokine imbalance (↑Leptin, ↓Adiponectin) and cytokine release (e.g., TNF-α, IL-6). (2) Systemic dissemination of inflammatory mediators and immune cell dysregulation. (3) Remodeling of the lung tumor microenvironment (TME) towards an immunosuppressive state, characterized by T cell exhaustion, expansion of immunosuppressive cells (myeloid-derived suppressor cells [MDSCs], regulatory T cells [Tregs], M2-polarized tumor-associated macrophages [TAMs]), and metabolic competition. (4) The dual impact on immune checkpoint inhibitor (ICI) outcomes: potential modulation of efficacy (via mechanisms underlying the “obesity paradox”) and increased risk for specific immune-related adverse events (irAEs), particularly endocrine toxicities.

Obesity-related systemic inflammation is a complex pathophysiological process that originates from the dynamic expansion and dysfunction of white adipose tissue (WAT) (13). This process is far from a simple increase in volume and represents a cascading response from cells to tissues and systems. Excessive energy surplus leads adipocytes to hypertrophy beyond the normal capillary’s range of oxygen supply, stabilizing and activating hypoxia-inducible factor-1α (HIF-1α) (14). As a critical transcription regulator, HIF-1α not only upregulates angiogenesis-promoting genes like vascular endothelial growth factor (VEGF) (15) but importantly enhances the recruitment capacity for pro-inflammatory immune cells like monocytes. At the same time, the overloaded state leads to endoplasmic reticulum stress and severe mitochondrial dysfunction within the cells, generating large amounts of reactive oxygen species (ROS) (16, 17), which not only directly damage cell structures but also participate as crucial signaling molecules in activating inflammatory pathways. These cellular stress signals converge, eventually activating two core inflammation signaling hubs—the NF-κB pathway and the JNK pathway (18). The activation of the NF-κB pathway, which is a “main switch” of the inflammation response, promotes the transcription and expression of numerous pro-inflammatory genes such as TNF-α, IL-6, and IL-1β upon exposure to stress signals like TNF-α or ROS (16, 18). The JNK pathway enhances the inflammatory reaction by phospholating transcription factor c-Jun (19), synergistically with NF-κB. The infiltration and polarization of immune cells play crucial roles in amplifying inflammation. The chemotactic gradient formed by MCP-1 (20) released by hypoxic environments and adipocytes recruiting monocytes into adipose tissue, where these monocytes polarize into M1-type macrophages with a strong pro-inflammatory nature, marked by high expression of CD11c (21) and significant production of TNF-α, IL-6, and IL-12 (22). Notably, these activated M1 macrophages typically surround necrotic adipocytes, forming characteristic “crown-like structures (23)”, exacerbating local inflammation when persistently activated. Furthermore, other immune cells such as CD8^+^T cells and Th1 cells infiltrate early in obesity, driving macrophages to M1 polarization (21, 24); conversely, anti-inflammatory Tregs, Th2 cells, and M2-type macrophages (23–25) are relatively reduced, leading to severe imbalance in the entire inflammatory regulation. The imbalance in adipokine spectra acts as a direct promoter of systemic metabolic disorder. Leptin, a hormone specifically secreted by adipocytes (26), correlates positively with body fat stores and promotes differentiation towards pro-inflammatory Th1 phenotype in CD4^+^T cells, significantly inhibiting Tregs function (23, 25). In contrast, adiponectin levels, which are supposed to have significant anti-inflammatory and insulin-sensitizing effects, decrease strikingly under obesity (27). This “high leptin-low adiponectin” imbalanced configuration forms the hormonal basis of obesity-related immunometabolic disorder (**Figure 1**).

### 2.2 Impact of obesity on the occurrence and development of lung cancer

Long hampered by the confounding factor of smoking, the epidemiological association between obesity and lung cancer risk is complex. However, large-scale meta-analysis (28) in recent years suggests a positive correlation between obesity and increased risk of lung adenocarcinoma in never-smokers. The underlying biological mechanisms of this association are multifaceted and involve the interaction of multiple systems. Hormonally, hyperinsulinemia associated with obesity can directly activate insulin receptors (IR) (29) and IGF-1 receptors (IGF-1R) (30) on tumor cell surfaces, strongly promoting protein synthesis, cell cycle progression, and inhibiting apoptosis through the core cell proliferation signal pathway PI3K-Akt-mTOR (31). Additionally, increased aromatase activity in adipose tissue leads to heightened conversion of androgens to estrogens, elevating local and systemic estrogen levels which possibly promote cell cycle progression and tumor angiogenesis in lung cancer cells through interaction with ER-β (32). Metabolically, adipose tissue releases large amounts of free fatty acids (FFAs) (33), serving not only as energy substrates but important signaling molecules, acting as natural ligands for PPARs (33, 34), regulating the expression profile of genes related to lipid metabolism and cell proliferation. FFAs can also activate the NF-κB pathway through TLR4 (34, 35), further intensifying local and systemic inflammation. On the inflammatory front, persistent systemic inflammation caused by obesity, through key signal axes like IL-6/JAK/STAT3 (36, 37), creates a highly favorable microenvironment for tumor occurrence and progression. Persistent STAT3 activation upregulates anti-apoptotic proteins like Bcl-2, survival proteins like Bcl-xL, and cell cycle proteins like Cyclin D1 (38, 39), facilitating tumor cell survival and proliferation. These interlinked, multi-level mechanisms define a complex network through which obesity influences lung cancer development.

### 2.3 Obesity’s role in shaping an immunosuppressive tumor microenvironment

**Figure 2:**
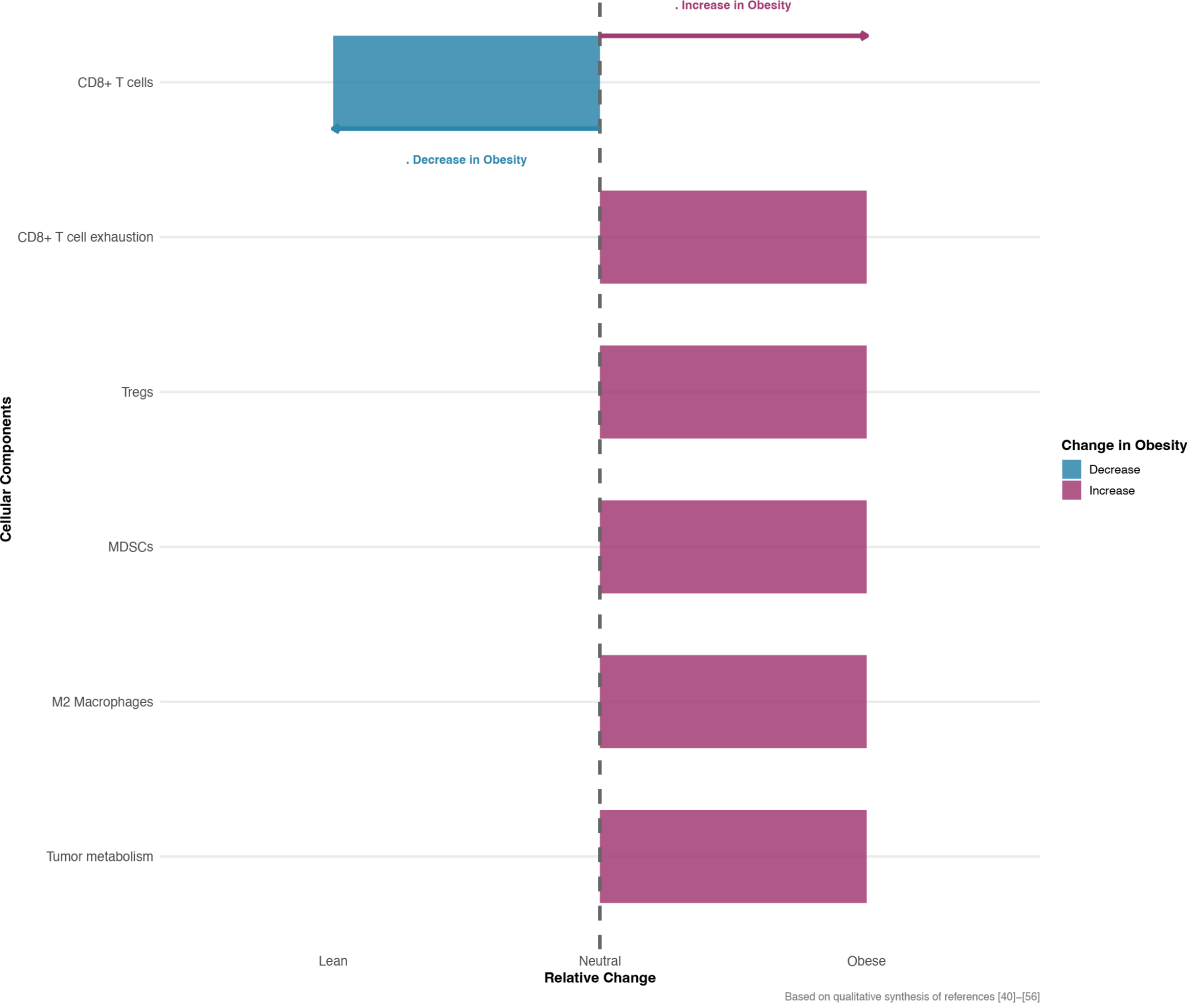
Schematic representation of obesity-induced changes in the lung tumor microenvironment (TME). This schematic illustrates the directional changes in key cellular components within the lung tumor microenvironment (TME) in obese compared to lean states, based on a qualitative synthesis of evidence from the literature (references 40–56 in the manuscript). Blue bars indicate a decrease, while red bars indicate an increase in the frequency or activity of each component in obesity. The T-cell exhaustion marker category refers to expression levels of inhibitory receptors (e.g., PD-1, TIM-3). MDSCs, myeloid-derived suppressor cells; Tregs, regulatory T cells. The figure was generated in R (v4.3.1) using ggplot2.

Obesity significantly restructures the immune landscape of lung cancer, favoring an overall immunosuppressive state. At the T cell functional level, CD8^+^T cells in the obese tumor microenvironment (TME) (40) not only have high expressions of immune checkpoint molecules such as PD-1, TIM-3, and LAG-3 (40, 41), but their metabolic adaptability is also severely impaired. Elevated levels of circulating leptin and insulin compel T cells towards lipid metabolic reprogramming, yet excessive lipid uptake and oxidation may induce lipotoxicity (42), leading to endoplasmic reticulum stress and mitochondrial dysfunction, ultimately promoting T cell exhaustion and apoptosis. Additionally, severe glucose deficiency in the TME, due to fierce competition for glucose between tumor cells and inhibitory immune cells (e.g., MDSCs), inhibits effective glycolysis in CD8^+^T cells, diminishing their energy supply for effector functions (43). Obesity also promotes the expansion of multiple immunosuppressive populations. Under the influence of obesity-related inflammatory cytokines like GM-CSF and IL-6, myeloid-derived suppressor cells (MDSCs) (44, 45) are abundantly generated and expanded in the bone marrow. These cells inhibit T cell function by secreting arginase-1 (Arg-1) and inducible nitric oxide synthase (iNOS) (46, 47), depleting the necessary amino acid arginine required for T cell proliferation and producing NO, which suppresses T cell receptor signaling. Regulatory T cells (Tregs) (48), whose differentiation and stability are enhanced in TME by TGF-β, IL-10, and oxidized lipids from FFAs (such as PGE2) (49, 50), directly inhibit effector T cell activation and functions through cell contact-dependent mechanisms (e.g., CTLA-4 depriving CD80/CD86 costimulatory signals (51)) and secretion of inhibitory cytokines. M2-type tumor-associated macrophages (TAMs) (51, 52), driven to M2 phenotype by IL-4, IL-13, and metabolites like lactate in the obese TME (51–53), support tumor angiogenesis via VEGF, tumor invasion, and metastasis via TGF-β, IL-10, and matrix metalloproteinases (MMPs) (51, 54–56), and directly suppress T cell antitumor functions. These changes collectively forge a highly immunosuppressive tumor microenvironment. (**Figure 2**).

### 2.4 The gut microbiota: intermediate mediator in obesity-inflammation-immunotherapy

**Figure 3:**
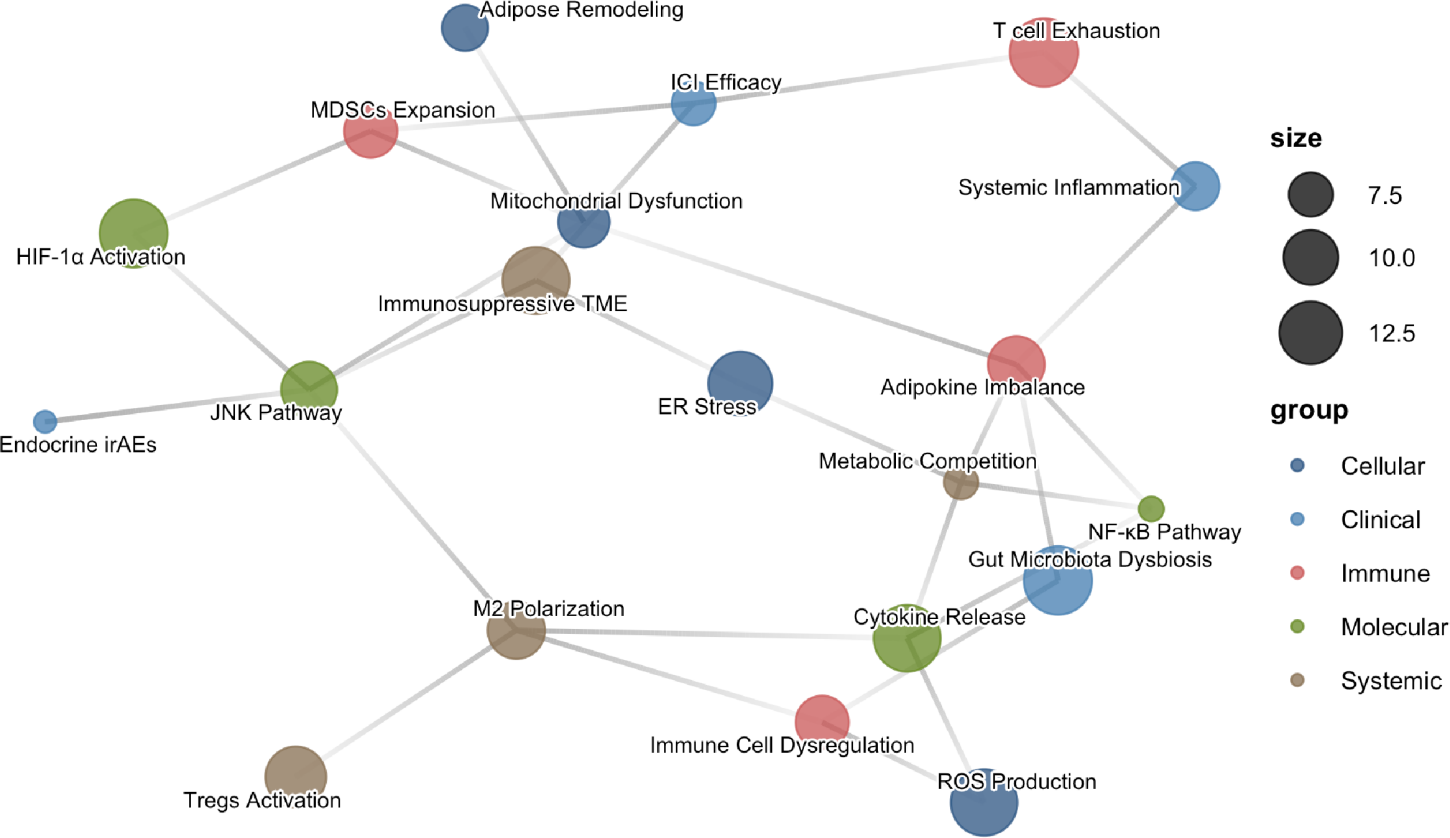
A multi-level network depicting obesity-related mechanisms impacting immune checkpoint inhibitor therapy in lung cancer. This network diagram visualizes the complex, interconnected biological processes linking obesity to the efficacy and toxicity of immune checkpoint inhibitor (ICI) therapy in lung cancer. Nodes represent distinct pathophysiological mechanisms, color-coded by their primary level of action: Cellular (blue), Molecular (orange), Immune (green), Systemic (purple), and Clinical (brown). Edges (connecting lines) denote documented directional influences, regulatory relationships, or strong associative pathways derived from the literature review. Node size is proportional to its computed centrality within the network, reflecting its relative importance in connecting different mechanistic layers. Key pathways illustrated include adipose tissue remodeling, endoplasmic reticulum (ER) stress, activation of inflammatory signaling (NF-κB, JNK), immune cell dysregulation (T cell exhaustion, expansion of MDSCs, Tregs, and M2 macrophages), gut microbiota dysbiosis, and their convergence on clinical outcomes such as ICI efficacy and endocrine immune-related adverse events (irAEs). This conceptual network was constructed and plotted using the igraph package in R.

As a complex “virtual endocrine organ”, disturbances in the gut microbiota are pivotal in linking obesity with immunotherapy response (57). Obesity-related dysbiosis is characterized by reduced microbial α diversity, increased Firmicutes/Bacteroidetes ratio, and significant reduction in beneficial bacteria like *Akkermansia muciniphila* and *Faecalibacterium prausnitzii* known for anti-inflammatory and gut barrier protective functions (58, 59). This dysbiosis influences host immune status and immunotherapy response through various mechanisms. Metabolic endotoxemia is a significant pathway whereby dysbiosis leads to reduced expression of tight junction proteins like Occludin and ZO-1 (60, 61) in the intestinal epithelium, compromising barrier integrity and allowing lipopolysaccharides (LPS) from the cell walls of Gram-negative bacteria to translocate into the portal circulation (62). These LPS activate TLR4 receptors on immune cells, triggering a systemic low-grade inflammatory state. In the realm of metabolic regulation, short-chain fatty acids (SCFAs) (62, 63) like butyrate and propionate, produced by microbiota fermenting dietary fibers, exert complex immunomodulatory effects. Butyrate promotes regulatory T cell differentiation to maintain immune tolerance through histone deacetylase (HDAC) (64, 65) inhibition; however, in tumor immunity contexts, such effects might inappropriately dampen antitumor immune responses. Reduced SCFAs production under obesity could disrupt this delicate balance (66). Systemically, specific beneficial microbial communities enhance the maturation of dendritic cells (DCs) (67), making them more effective in antigen presentation and IL-12 secretion, which fosters T cell differentiation into Th1 and cytotoxic T cells (68). Akkermansia muciniphila, for instance, has been shown to enhance DC recruitment and activation of CD4^+^T cells (69, 70). Obesity-related dysbiosis, however, might weaken this process, affecting the host’s response to ICIs (**Figure 3**).

## 3 Obesity and ICI efficacy: from “paradox” to mechanism

### 3.1 The reappearance and controversy of the “obesity paradox” in lung cancer immunotherapy

The evidence regarding the “obesity paradox” in lung cancer ICI therapy shows significant inconsistency, deeply reflecting its complex biological background and methodological issues (71). Interpretation of this paradox is inherently limited by the observational nature of most supporting studies, which are susceptible to reverse causation, selection bias (e.g., healthier obese patients being more likely to receive and tolerate full ICI courses), and residual confounding from unmeasured factors like physical activity, diet, or concomitant medications (45, 67, 72). Several large retrospective cohort studies have reported associations between high BMI and improved survival with ICIs. For instance, a pooled analysis of clinical trials by Kichenadasse et al. (73) found that in NSCLC, melanoma, and renal cancer patients, overweight/obesity (BMI ≥25 kg/m²) was associated with significantly improved overall survival (OS) (HR ~0.65-0.85, depending on cancer type) compared to normal BMI. The underlying hypothesis is that obese patients may possess a higher tumor mutation burden (TMB) and enhanced T cell activation mediated by leptin (74), potentially providing advantages for immunotherapy (75). However, studies challenging this paradox continue to emerge, such as a study (76) highlighting a negative correlation between BMI and objective response rate (ORR): Krejčí et al. (76) found no significant association between BMI and PFS/OS in their NSCLC cohort. Another study (77) focusing on East Asian NSCLC patients found that higher BMI correlated with poorer progression-free survival (PFS), suggesting an influential role of racial and fat distribution differences—where Caucasians tend to have more subcutaneous fat versus East Asians who accumulate more visceral fat, associated with worse metabolic and inflammatory states. Key factors explaining these heterogeneities include treatment plan variability, where the paradox might be more evident in ICI monotherapy as chemotherapy’s cytotoxic effects might obscure or alter obesity’s impacts in combined ICI treatments (78); the backgrounds of tumor driver genes are critical as well—in EGFR-mutated (79) or ALK-fusion lung cancers (80), the impact of obesity on ICI efficacy could be drastically different given their innate unique immune microenvironments such as low T cell infiltration; and limitations in the BMI metric itself shouldn’t be overlooked; it cannot distinguish sarcopenic obesity, a phenotype associated with the worst prognosis. A normal-BMI patient might be sarcopenic due to significant muscle loss, whereas a high-BMI patient might possess adequate muscle mass, directly affecting study outcomes interpretation (77, 80, 81).

### 3.2 Exploration of potential biological mechanisms from multiple angles

From the “fuel” hypothesis perspective, indeed obesity might elevate TMB via chronic inflammatory states (82), but high TMB alone isn’t an absolute guarantor of immunotherapy efficacy. More crucially, the “fuel” provided by adipose tissue exhibits a marked duality: while free fatty acids can be utilized by activated T cells for β-oxidation to sustain their long-term memory functions, in dysfunctional tumor microenvironments, excessive lipid uptake by CD8^+^T cells could lead to lipid overload, inducing T cell exhaustion (82, 83). Recent studies (84, 85) have discovered that exhausted T cell precursors (TCPs) present unique lipid metabolic profiles, suggesting close ties between lipid metabolism and T cell fate decisions. Concerning metabolic activation versus functional impairment, leptin’s dose-response relationship in T cell activation is complex; physiological levels of leptin are imperative for T cell survival and function; prolonged exposure to supraphysiological levels of leptin in obesity (85, 86), however, may lead to leptin resistance, akin to insulin resistance, eventually resulting in T cell dysfunction, possibly involving upregulation of SOCS3 protein (86), inhibiting the JAK-STAT pathway through negative feedback mechanisms (86, 87). From a pharmacokinetic (PK) consideration standpoint, early treatment plans for ICIs like Nivolumab that dose by weight theoretically pose underexposure risks for obese patients (88, 89). However, ICI action is receptor-mediated, displaying complex PK/PD (pharmacokinetic/pharmacodynamic) relationships. Most modern ICIs adopt fixed dosing; population pharmacokinetic models show negligible clinical significance in exposure differences across weight groups (90). However, whether body composition (such as muscle and fat proportions) impacts drug distribution volume and clearance rate remains to be elucidated through more precise research. The impact of sex dimorphism should not be ignored; estrogen modulates immune responses via various mechanisms such as enhancing dendritic cell antigen presentation functionality and adjusting B cell responses (91); in premenopausal women, heightened estrogen levels might offset some negative inflammatory effects of obesity (92), partly explaining the discrepancy in “obesity paradox” manifestation among different genders.

### 3.3 Beyond BMI: the era of body composition analysis

**Figure 4:**
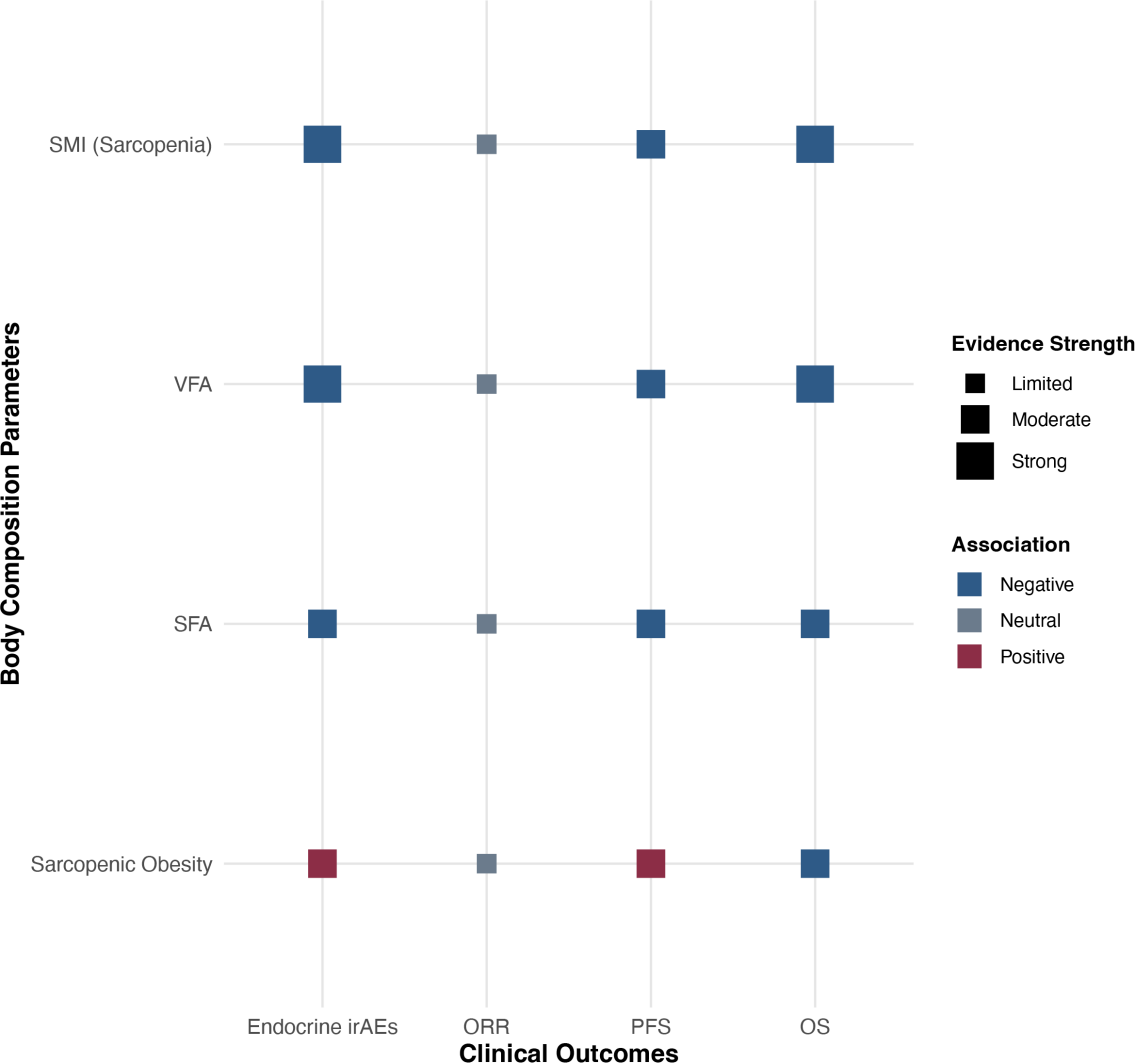
Qualitative associations between body composition parameters and clinical outcomes of immune checkpoint inhibitor (ICI) therapy in lung cancer. This dot plot summarizes reported associations between body composition parameters and ICI clinical outcomes, derived from a literature review (references 93–99). Association direction is indicated by color (blue: negative; gray: neutral/inconsistent; red: positive). Dot size represents the strength of supporting evidence across studies (small: limited; medium: moderate; large: strong). SMI, skeletal muscle index; VFA, visceral fat area; SFA, subcutaneous fat area; OS, overall survival; PFS, progression−free survival; ORR, objective response rate; irAEs, immune−related adverse events. The figure was generated in R (v4.3.1) using ggplot2.

Sarcopenia, as a progressive and widespread loss of skeletal muscle mass and function reflecting systemic inflammation and malnutrition, is a key organ for protection and protein storage in insulin metabolism and playing an immune regulation role. In ICI therapy, low skeletal muscle index (SMI) (93) independently associates with poorer OS and PFS, potentially attributable to systemic inflammation’s consumption, probable impacts on ICI drug distribution volume, and diminished vital roles in antitumor immunity basis exerted by myokines such as IL-6, IL-7, and IL-15 (94). Sarcopenic obesity represents the “worst of both worlds,” characterized by dual features of muscle function loss and adipose inflammation burden. These patients often present the highest inflammatory markers (such as C-reactive protein) (95), the most severe insulin resistance, and the weakest antitumor immune response. Its diagnosis heavily relies on precise body compositions analysis; BMI alone might completely miss diagnosing such high-risk groups. Different fat distributions also play decisive roles: visceral adipose tissue (VAT) (96) is highly metabolically active, its inflammation factor secretions directly enter the liver through the portal vein, being the main source of whole-body inflammation; high visceral fat area (VFA) (97) clearly links with poorer PFS and higher immune-related adverse event (irAEs) risks; subcutaneous adipose tissue (SAT) relatably less active metabolically, perhaps serving as a “safe” energy repository (98), preventing lipid ectopic deposition in viscera and muscles, and some studies found higher SFA correlates with better prognoses or at least presents neutral impacts; intermuscular fat, emerging as a “fat quality” indicator, closely correlates with insulin resistance and body functional decline (99). These detailed body composition analysis metrics promote us towards transcending simple BMI evaluations, advancing into a new era of precisely assessing the relationships between obesity and immunotherapy (**Figure 4**).

## 4 Obesity and immune-related adverse events: focus on endocrine toxicity

### 4.1 The general framework of obesity as an immune toxicity risk modulator

Obesity-associated chronic, low-grade inflammatory states form a “pre-activated” immune context, affecting not only the efficacy of ICIs but possibly altering the risk spectrum and severity of irAEs (98, 99). Theoretically, in baseline obese patients with an immune system above vigilance state, changing the balance point of immune homeostasis might lower the threshold for irAEs, causing more propensity for immune systems to overly activate and attack normal tissues once ICIs lift the brakes, especially those organs already closely related to metabolism and inflammation like endocrine glands.

However, clinical studies don’t uniformly support this. Studies (100–102) involving NSCLC patients receiving ICI indicated no significant statistical correlation between BMI and overall irAEs risk; yet, overweight or obese patients faced markedly increased risks of endocrine irAEs. This suggests obesity might selectively affect specific types of irAEs rather than universally amplifying all types of toxicity risks.

### 4.2 The unique correlation between endocrine irAEs: mechanisms and clinical aspects

#### 4.2.1 Mechanisms with high concordance

There exists a profound pathophysiological connection between obesity and endocrine irAEs. First, a “cross-talk” tightly exists between adipose tissue itself and endocrine glands (such as pituitary, thyroid, pancreas) (103), sharing multiple receptors and signaling pathways. Second, adipocytes and immune cells can jointly express some immune checkpoint molecules like PD-L1 and CTLA-4 (1), possibly making endocrine organs common immune attack targets. Third, leptin directly regulates the hypothalamic-pituitary axis (such as controlling TSH and ACTH secretion) (104), with hyperleptinemia potentially heightening the hypothalamus and pituitary’s sensitivity to immune attacks. Lastly, the molecular mimicry theory posits that adipocytes may share similar antigenic epitopes with certain endocrine cells (like pancreatic β cells) (105), causing cross-immunity reactions (**Table 1**). These interactions are integrated into the broader mechanistic network shown in **Figure 3**.

**Table 1:** Potential interaction mechanisms between obesity and endocrine toxicities associated with immune checkpoint inhibitors.

Mechanism	Specific processes	Potential consequences
Adipokine Imbalance	Significantly increased leptin levels and decreased adiponectin levels.	Promotes systemic inflammation, alters T-cell and macrophage function, and breaks immune tolerance.
Inflammatory Cytokine Storm	Sustained release of pro-inflammatory cytokines (e.g., TNF-α, IL-6, IL-1β) into the bloodstream.	Lowers the threshold for immune tolerance, increases vascular permeability, and promotes immune cell infiltration into endocrine organs.
Immune Cell Infiltration and Activation	Increased M1 macrophages and cytotoxic T cells in adipose tissue, accompanied by impaired Treg function.	Enhances immune surveillance of endocrine organs and the capacity for specific attacks against them.
Metabolic Dysregulation Background	Presence of insulin resistance and elevated free fatty acids (FFA).	Directly affects the function and survival of endocrine cells (e.g., pancreatic beta cells), increasing their vulnerability to stress.
Molecular Mimicry and Cross-Reactivity	Potential sharing of antigenic epitopes between adipocytes and endocrine cells (e.g., thyroid follicular cells, pituitary cells).	Activated T-cells targeting tumor antigens may mistakenly damage endocrine tissues expressing similar antigens.

#### 4.2.2 Clinical evidence review and case analysis

Thyroid dysfunction is the most common endocrine irAE. Studies consistently show an increased risk of ICI-related thyroiditis (often presenting as transient thyrotoxicosis followed by persistent hypothyroidism) in obese patients. For instance, in the case reported by Rossi et al. (106), a 39-year-old male patient with BMI 32 kg/m² experienced palpitations, excessive sweating, and symptoms of thyrotoxicosis just three weeks after receiving pembrolizumab, with thyroid function tests showing TSH suppression, elevated FT3/FT4, and diffusely inflamed thyroid ultrasound. Symptoms swiftly progressed to hypothyroidism within weeks, requiring lifelong levothyroxine replacement therapy.

Pituitary inflammation is another common and serious endocrine irAE, more frequent in patients receiving CTLA-4 inhibition (especially ipilimumab) or combination therapies. Obese patients, especially those with central (android) obesity, likely predisposed by altered baseline HPA (hypothalamic-pituitary-adrenal) axis state (e.g., mildly elevated cortisol levels) (107), may be more susceptible to pituitary inflammation. In the aforementioned case by Rossi (106), the patient showed acute symptoms of pituitary inflammation three months into pembrolizumab treatment, including high fever, severe dizziness, intense headache, and hyponatremia, with lab tests revealing extremely low ACTH and cortisol levels, confirming secondary adrenal insufficiency necessitating immediate intravenous glucocorticoid supplementation.

Significantly, the case developed an unusual transition from secondary adrenal insufficiency to primary adrenal insufficiency a year after ICI treatment, with lab tests showing a shift from low ACTH/low cortisol to high ACTH/low cortisol, requiring a switch from glucocorticoid-only supplementation to combined glucocorticoid and mineralocorticoid replacement (106). This rare, dynamically evolving endocrine toxicity spectrum strongly indicates obesity potentially affecting the process and pattern of endocrine organ damage, possibly involving ongoing autoimmune attacks spreading to more glands.

Type 1 diabetes is relatively rare yet potentially life-threatening among endocrine irAEs, often debuting as diabetic ketoacidosis (DKA) (108). Tang et al. (109) reported a case of a 61-year-old female lung adenocarcinoma patient with a BMI of 28.5 kg/m², diagnosed with diabetes after four months of pembrolizumab and suffering repeated severe DKA episodes later in the disease course. Lab testing revealed high titers of glutamic acid decarboxylase antibodies (GADAb) and very low C-peptide levels, confirming ICI-induced type 1 diabetes. For obese patients, baseline insulin resistance and β cell functional compensatory hyperactivity complicate and make the onset, diagnosis, and glycemic management of diabetes more challenging and thorny.

Additionally, primary adrenal insufficiency and hypoparathyroidism are less common endocrine irAEs. Still, sporadic case reports (110) suggest obese patients might face higher risks, possibly associated with complex hormonal interactions between adipose tissue and these glands.

### 4.3 Challenges and optimization strategies in managing irAEs against an obesity background

Diagnosing endocrine irAEs among obese patients uniquely challenges. First, common obesity symptoms (like fatigue, lethargy, appetite changes, cold/heat intolerance) heavily overlap with symptoms of thyroid dysfunction or hypopituitarism, easily overlooked or mistaken for treatment-related fatigue or tumor progression (92, 107). Second, obesity often accompanies baseline hormone level physiological changes, such as mildly elevated cortisol, reduced SHBG impacting sex hormone level assessments (111), all interfering with endocrine function interpretations.

In terms of treatment, challenges are as prominent. The utilization of high-dose glucocorticoids (like prednisone 1–2 mg/kg/day) (112) necessary for severe endocrine irAEs (like severe hypophysitis) poses metabolic burden risks in obese patients, including further weight gain, abnormal glucose tolerance or overt diabetes, exacerbated hypertension, and hyperlipidemia (113). Thus, management strategies demand higher fineness: whilst ensuring effective inflammation control, the aim should be to minimize high-dose steroid usage duration, transfer to maintenance low-dose hormone replacement therapy early (like levothyroxine for hypothyroidism, hydrocortisone for adrenal insufficiency). Meanwhile, proactive initiation or strengthening drug therapy tackling obesity comorbidities, like employing metformin or SGLT2 inhibitors for glycemic control, is crucial.

Multidisciplinary cooperation (MDT) management is essential for these patients. Core collaboration between oncology and endocrinology is the bedrock for precise diagnosis and effective management of endocrine irAEs. Furthermore, early involvement of nutrition departments helps devise personalized anti-inflammatory diet plans, controlling weight and stabilizing metabolism; pharmacy departments participation optimizes drug selection and doses, managing drug interactions; when necessary, rehabilitation medicine guides safe and effective exercise to improve body composition and physical status. Comprehensive care is crucial to ensure obese lung cancer patients can safely endure continued immunotherapy, maximizing survival benefits.

## 5 Construction of novel biomarkers and predictive models

### 5.1 Blood-based biomarkers

Circulating adipokines are the most direct fluid reflection of obesity-related inflammation, holding massive predictive potential. Studies (114, 115) show that the leptin/adiponectin ratio is a more stable predictor than individual markers, with higher ratios often signifying more substantial inflammatory states and poorer ICI therapy outcomes. Additionally, systemic inflammation indicators derived from complete blood counts, such as neutrophil-lymphocyte ratio (NLR) (116), platelet-lymphocyte ratio (PLR) (117), and systemic immune-inflammation index (SII = platelet × neutrophil/lymphocyte) (118), often significantly elevate in obese patients and have been confirmed by numerous studies associating with poorer immunotherapy OS and PFS.

Soluble immune checkpoint molecules like sPD-L1, sCTLA-4, alongside classical inflammatory cytokines (IL-6, TNF-α, CRP) (119, 120) level changes in serum of obese patients, might also provide additional predictive value. Collectively, these factors reflect systemic immune activation states and inflammatory burden, aiding in identifying patients who might benefit from more proactive inflammation management, closer toxicity surveillance, or alternate combination therapy strategies before treatment.

### 5.2 Imaging-based biomarkers

The conventional CT scan used for therapeutic efficacy evaluation remains an unexploited biomarker treasure house. With specific semi-automated software (e.g., Slice-O-Matic) (121) analyzing body composition at the third lumbar vertebra (L3) level, parameters such as skeletal muscle index (SMI), visceral fat area (VFA), and subcutaneous fat area (SFA) (122) can be accurately quantified, bypassing BMI and offering a more precise obesity assessment (**Table 2**). .

**Table 2:** Predictive value of imaging-based body composition parameters for ICI efficacy and toxicity.

Parameter	Measurement method	Predictive value	Limitations
Skeletal Muscle Index (SMI)	Total cross-sectional area of skeletal muscles (e.g., psoas, erector spinae) at the L3 level, normalized to height squared (cm²/m²).	Low SMI (sarcopenia) is a strong, independent predictor of poorer Overall Survival (OS) and Progression-Free Survival (PFS) with ICIs.	Diagnostic cut-off points vary across different ethnicities, genders, and age groups.
Visceral Fat Area (VFA)	Cross-sectional area of the intra-abdominal fat compartment at the L3 level (cm²).	High VFA is associated with a higher overall risk of immune-related Adverse Events (irAEs), particularly endocrine and gastrointestinal toxicities, and potentially poorer PFS.	Measurement and delineation require expertise, and there is variability between software applications.
Subcutaneous Fat Area (SFA)	Cross-sectional area of the subcutaneous fat layer at the L3 level (cm²).	The relationship with prognosis is inconsistent; most studies consider its impact neutral or slightly protective.	The clinical significance and optimal cut-off points are not well defined.
Sarcopenic Obesity	Combination of low SMI and high body fat percentage or high VFA.	One of the strongest negative predictors; patients with this phenotype typically have the worst OS and PFS.	Requires specialized software for analysis and has not yet been incorporated into routine clinical practice.

Radiomics, extracting a vast number of quantitative features (e.g., texture, shape, wavelet features) from medical images (CT, PET-CT), captures intratumoral heterogeneity and microstructural information invisible to the naked eye. Studies (123, 124) demonstrate that radiomic features based on baseline CT, combined with body composition analysis, can construct more potent models than singular metrics for predicting primary resistance (125), secondary resistance, and risk of specific irAEs to ICIs. Moreover, PET-CT provides metabolic information like standardized uptake values (SUV) (126) of adipose tissue or skeletal muscle, potentially reflecting their inflammatory activity, adding extra dimensions for predictions.

### 5.3 Integrative multiomics data predictive models

**Figure 5:**
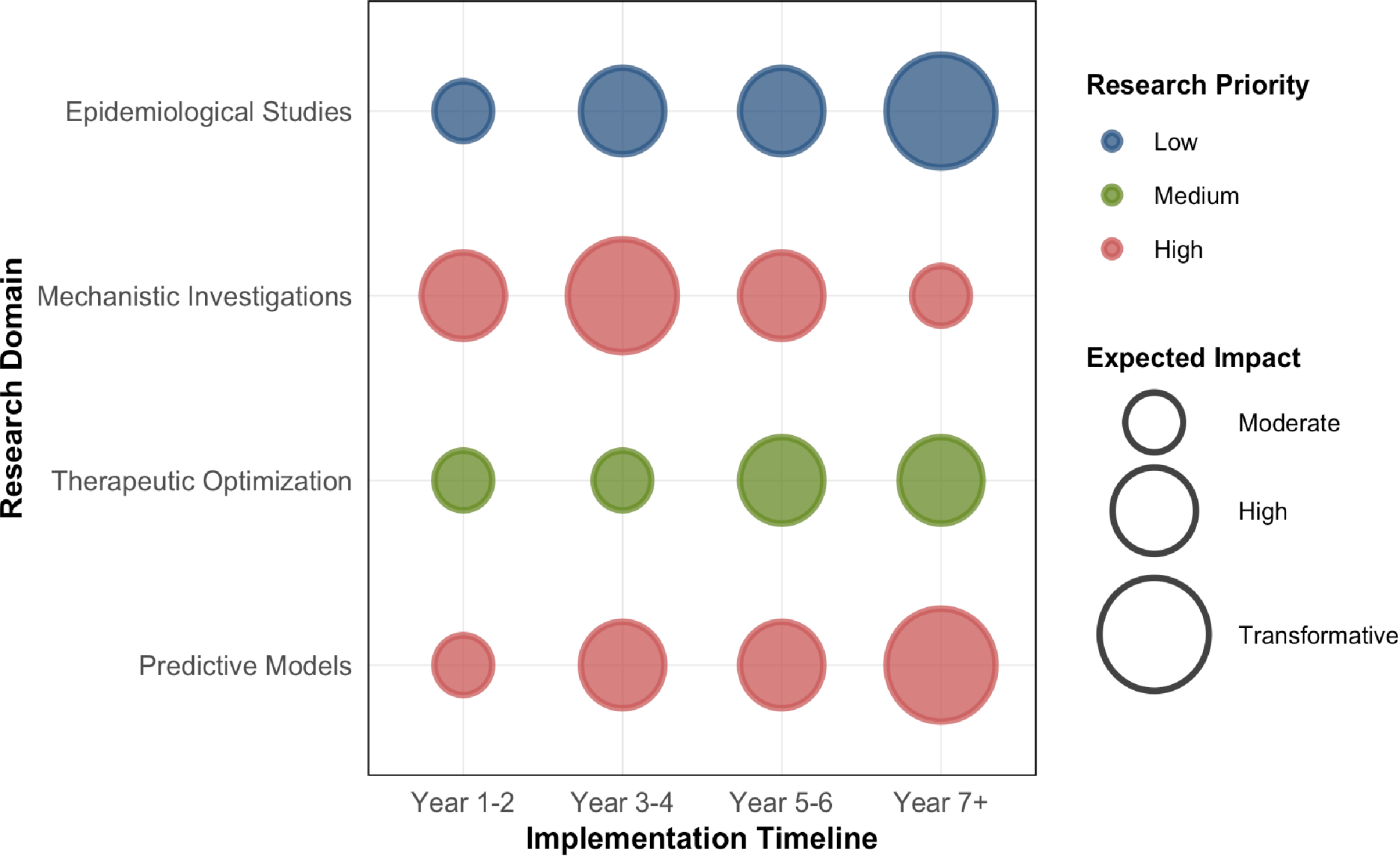
Strategic research roadmap for tool development. This bubble chart presents a strategic roadmap for research domains (y-axis) across implementation timelines (x-axis). Bubble size represents the expected scientific impact (Moderate: small; High: medium; Transformative: large). Bubble color indicates research priority (Low: blue; Medium: orange; High: green). High-priority initiatives include: development of predictive models integrating multi-modal data, therapeutic optimization strategies, mechanistic investigations using single-cell omics, and large-scale epidemiological studies with detailed body composition analysis.

Single biomarkers fail to comprehensively capture the intricacies of obesity, inflammation, and immune therapy response. Therefore, research shifts towards developing predictive models integrating multiomics datasets. Machine learning and AI algorithms manage high-dimensional data, identify complex patterns, and establish more accurate predictive models. These models can integrate clinical features (e.g., BMI, age, sex), lab parameters (e.g., adipokines, inflammatory markers), imaging features (e.g., body composition, radiomic features), and traditional biomarkers (e.g., PD-L1 expression, TMB), generating comprehensive predictive scores. Additionally, some research explores incorporating gut microbiomes into predictive models due to the close association between gut microbiota, obesity, and immune therapy responses (127). Despite promising outlooks, clinical translation faces challenges like model validation, standardization, and generalizability. Future large-scale prospective studies must verify model performance across diverse populations, determining their practical value in clinical decision-making (**Figure 5**, **Table 3**).

**Table 3:** Summary of key biomarkers and their clinical readiness.

Biomarker category	Specific examples	Potential clinical utility	Current readiness for clinical use
Adipokines	Leptin, Adiponectin, Leptin/Adiponectin Ratio	Predict systemic inflammation burden, ICI efficacy, and irAE risk.	Experimental. Require standardized assays and validated cut-offs in large prospective cohorts. Not yet routine.
Systemic Inflammatory Indices	NLR, PLR, SII, CRP	Readily available from routine blood tests; predict OS/PFS and inflammation status.	Near-term potential. Easily implementable but need consensus on optimal cut-offs and integration into decision algorithms.
Imaging Body Composition	SMI (Sarcopenia), VFA, SFA, Sarcopenic Obesity	Provide direct quantification of muscle and fat depots; strong prognostic value for efficacy/toxicity.	Translational. CT-based analysis requires specialized software and expertise. Standardization of measurement protocols and diagnostic criteria across populations is needed before widespread adoption.
Radiomics	Texture, shape, wavelet features from CT/PET-CT	Capture intratumoral heterogeneity and microenvironment features; may predict primary/secondary resistance and specific irAEs.	Early research. Promising but faces challenges in feature reproducibility, model validation, and generalizability. Not ready for clinical use.
Gut Microbiota	Microbial diversity, specific taxa (e.g., Akkermansia), metagenomic signatures	May predict ICI response and modulate irAEs via immune-metabolic axes.	Preclinical/Early clinical. Highly complex and variable. Fecal microbiota transplant (FMT) and probiotics are being tested in trials. Far from routine clinical application.
Multiomics Integrative Models	Combined clinical, imaging, genomic, proteomic, microbiome data via AI/ML	Holistic prediction of efficacy and toxicity, enabling personalized strategies.	Future direction. Requires massive, standardized datasets, robust computational infrastructure, and rigorous prospective validation. Represents the ultimate goal but not yet clinically available.

## 6 Clinical management strategies and future prospects

### 6.1 Overall strategy for comprehensive, precise management of ICI therapy in obese lung cancer patients

Effective ICI therapy for obese lung cancer patients requires a continuous, multi-dimensional, individualized management strategy throughout pre-, during, and post-treatment phases. A comprehensive baseline assessment, far more than standard checks, is needed pre-treatment. This includes endocrine function evaluation (full thyroid panel, morning cortisol, ACTH, HbA1c or fasting glucose), precise body composition analysis (via diagnostic CT or bioelectrical impedance analysis BIA), and inflammation status assessment (e.g., complete blood count NLR calculation, CRP). These assessments aid in identifying high-risk patients pre-therapy (e.g., sarcopenic obesity, high VFA, baseline borderline endocrine function) to develop more targeted monitoring plans and patient education.

During treatment, enhanced interventions focused on endocrine irAEs proactive monitoring and screening should be implemented. According to guidelines from authoritative bodies like the European Society of Endocrinology (128), recommended frequency is baseline, before each treatment cycle, or at least every 4–6 weeks for systematic detection of TSH, free T4, morning cortisol, electrolytes (sodium, potassium, calcium), and glucose. For high-risk patients on combination immunotherapy (CTLA-4 plus PD-1/PD-L1 inhibitors) (129–131), pituitary hormones including ACTH, LH, FSH, estrogens (premenopausal women), testosterone (men), and prolactin should be considered. Patient education is critical; they must understand common endocrine irAEs symptoms (e.g., persistent fatigue, headache, visual changes, polyuria, palpitations) and be encouraged to report any new or persistent symptoms immediately.

Post-occurrence of endocrine toxicity requires dual focus: effectively controlling immune inflammation and meticulous management of metabolic sequelae. For severe endocrine irAEs, temporary ICI suspension and high-dose glucocorticoid induction might be required. For obese patients, steroids necessitate special caution, with close monitoring of weight, blood glucose, blood pressure, and lipid changes while taking preventative measures (e.g., prophylactic proton pump inhibitor use). Most cases allow for ICI therapy restart once the endocrine function stabilizes via hormone replacement therapy (like levothyroxine or hydrocortisone) (132); decisions should be collaboratively made by oncologists and endocrinologists.

### 6.2 Synergistic role of lifestyle interventions

Dietary adjustments and exercise as adjuncts have a growing import. For obese lung cancer patients, anti-inflammatory diet regimens like the Mediterranean diet, characterized by Omega-3 fatty acids (from fish), antioxidants (from colorful vegetables/fruits), and dietary fibers (from whole grains/legumes) (127, 133), while limiting refined sugars and saturated fats, are recommended. These dietary patterns may improve gut microbiota composition in obese patients, lower systemic inflammation, and potentially create a more favorable microenvironment for immunotherapy efficacy (134).

Exercise plays a multirole. Regular aerobic and resistance training not only helps augment skeletal muscle mass, reduce body fat (notably visceral fat) but also directly modulates immune function, like promoting T cell differentiation into more effector and memory phenotypes, enhancing antitumor immunity, potentially mitigating certain irAEs’ (e.g., fatigue) severity (135, 136). Designing personalized, progressive exercise plans for obese lung cancer patients stands as an essential non-pharmacological means to optimize body composition, improve treatment tolerance, and overall clinical outcomes.

### 6.3 Novel combination treatment strategies targeting the obesity-inflammation pathway

Combination therapy targeting the obesity-inflammation axis with ICIs represents a promising future research direction. Established drugs like anti-IL-6 antibody (Tocilizumab) and anti-TNF-α antibody (Infliximab) (137, 138) have success in severe irAEs treatment. Prospective studies explore the prophylactic or therapeutic use of these agents in specific high-risk obese patients (e.g., those with high inflammation markers (139)), aiming to alleviate toxicity while potentially enhancing efficacy.

Beyond glucose reduction, metformin has demonstrated anti-inflammatory and immune modulation properties, which might counteract obesity-related immunosuppression. SGLT2 inhibitors (e.g., dapagliflozin) (140, 141) effectively promote weight loss, enhance insulin sensitivity, and reduce blood pressure, warranting exploration with ICI combinations in obese lung cancer patients.

Leptin receptor antagonists, adiponectin receptor agonists or analogues development remains in preclinical or early clinical stages, but they offer fresh paths to directly intervene in obesity’s core pathology, optimizing immunotherapy strategies.

Finally, targeting obesity-related dysbiosis correction through specific probiotics, prebiotics, synbiotics, or microbiota transplant (FMT) (142) is an emerging topic. The clinically ‘tolerant’ gut microbiota has the potential to improve ICI efficacy and reduce irAE during the active trial phase.

### 6.4 Future research directions

Future research demands systematic, deeper investigation from translation-enabling perspectives into the complex engagement of obesity with lung cancer immunotherapy. The imperative first step is rigorous execution of large-scale prospective cohort studies beyond simple BMI stratifications, incorporating precise body composition metrics as core variables. Studies must adopt quantitative CT or MRI techniques for detailed skeletal muscle index, visceral and subcutaneous fat area measurement pre-, during, and post-treatment, establishing standardized measurement procedures and diagnostic cut-offs. Prospective study design must carefully consider racial differences, tumor molecular subtypes (e.g., EGFR mutations, KRAS mutations, STK11 deletions delineations) (143, 144), immune therapy plans (ICI monotherapy, ICI-chemo combos, ICI-angiogenesis inhibitors) (145), and treatment lines as key confounders. Research endpoints should transcend conventional survival metrics, focusing in-depth on unique immunotherapy patterns like duration of sustained response, incidence and nature of secondary resistance, and associations of pseudoprogression and hyperprogression (146–148). Equally important is the comprehensive characterization of irAEs, particularly endocrine toxicity, GI toxicity, and pneumonitis, detailing their timing, severity, and impact on treatment continuation. These irAE profiles should be utilized as efficacy-equivalent study endpoints to construct complete obesity-immunotherapy benefit-risk profiles.

Mechanistic level exploration plans must leverage cutting-edge technologies to unveil obesity’s biological foundation affecting immune therapy across dimensions. Molecularly, spatial transcriptomics and single-cell multiomic sequencing should refine phenotyping of immune, tumor, and stromal cells in obese versus non-obese lung cancer individuals (149), alongside visceral and subcutaneous fat, delineating their distinct gene expressions, epigenetic states, and metabolic characteristics to illustrate how obesity reshapes TMEs’ cellular ecology and intercellular communication networks down to single-cell resolution. Metabolically, research should center lipid metabolism-immune function cross-talk, probing how specified lipids (e.g., sphingolipids, oxidized phospholipids) modulate T cell, macrophage metabolic pathways, membrane fluidity, and signaling transduction during obesity to impact differentiation, function, and exhaustion states of immune cells (150). Additionally, explicating adipose-derived exosome roles in carrying specific miRNA, lipids, proteins for remote regulation of tumor immune responses is necessary. Systemically, an integrative biological research framework must cover host (genetic background, endocrine status, neuroendocrine regulation), gut microbiome (composition, functional gene, metabolites), and tumor (genome, immune microenvironment) (151), deciphering by systems biology methods how their dynamic interactions collectively determine the ultimate immune therapy outcome.

On the clinical tool development front, precise predictive model construction for clinical decision guidance is pivotal. Standardization of large-scale, multicenter, prospectively collected multiomic data is required, including clinical characteristics, laboratory checks, serial time-point imaging features, circulating biomarkers (e.g., ctDNA, cytokines, adipokines), and gut metagenomic data (152). Advanced machine learning algorithms like deep learning or ensemble learning should train integrative models capable of predicting efficacies (e.g., objective response, long-term survival) and specific toxicities (e.g., ≥grade 3 endocrine irAEs, immune pneumonitis) concurrently (153). Model outputs shouldn’t merely be simple risk scores but visualized, individualized benefit-risk probability maps capable of simulating different therapeutic strategies (single versus combined, varied monitoring frequencies) (154). Ultimately, validating these models’ effectiveness and practicability in real-world clinical decision-making, assessing their potential for genuinely improving patient life quality and longevity via prospective randomized controlled clinical trials is necessary (155).

**Figure 6:**
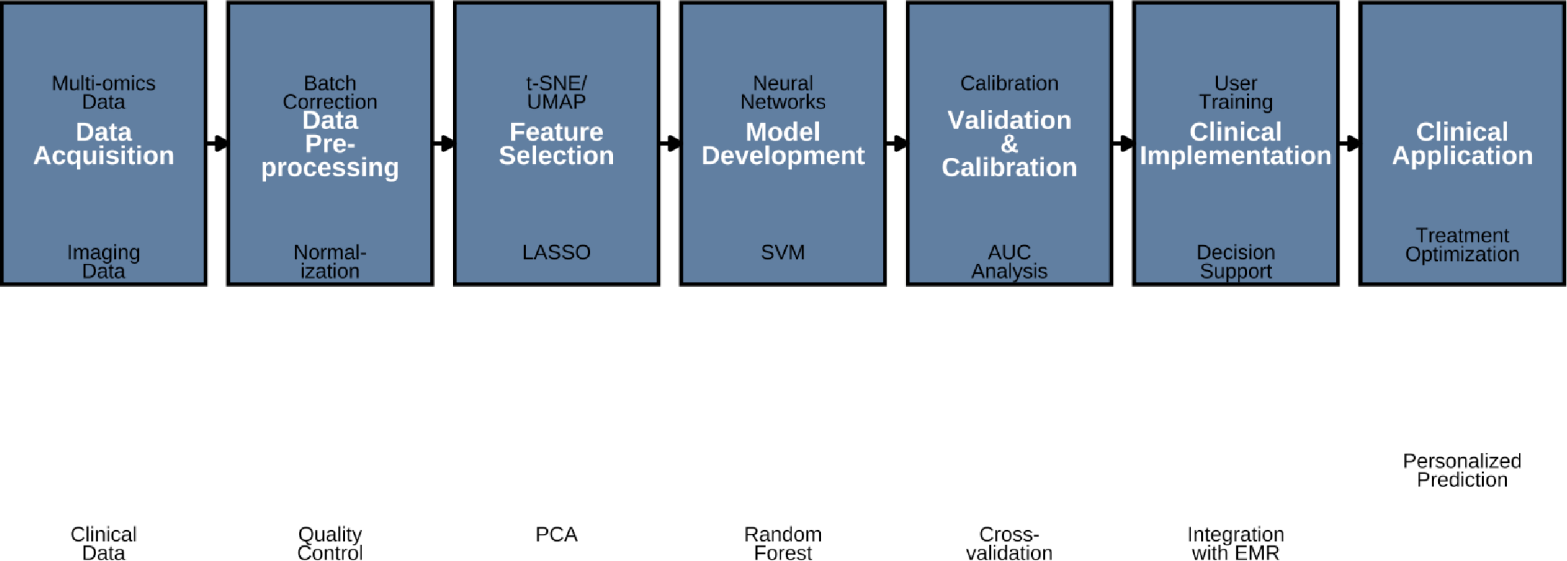
Workflow for multi-modal predictive model development. This process diagram outlines a comprehensive pipeline for developing multi-modal predictive models of ICI response in obese lung cancer patients. The workflow encompasses seven major stages (blue rectangles): (1) Multi-modal data acquisition from clinical, imaging, and multi-omics sources; (2) Data preprocessing including quality control, normalization, and batch correction; (3) Feature selection and dimensionality reduction using techniques like principal component analysis (PCA), least absolute shrinkage and selection operator (LASSO), and t-distributed stochastic neighbor embedding (t-SNE)/uniform manifold approximation and projection (UMAP); (4) Model development employing machine learning algorithms such as random forest, support vector machines (SVM), and neural networks; (5) Rigorous validation and calibration via cross-validation, area under the curve (AUC) analysis, and calibration curves; (6) Clinical implementation and integration with electronic medical records (EMR), decision support systems, and user training; (7) Final clinical application for personalized prediction and treatment optimization. Light green circles denote specific sub-processes within each stage.

**Figure 7:**
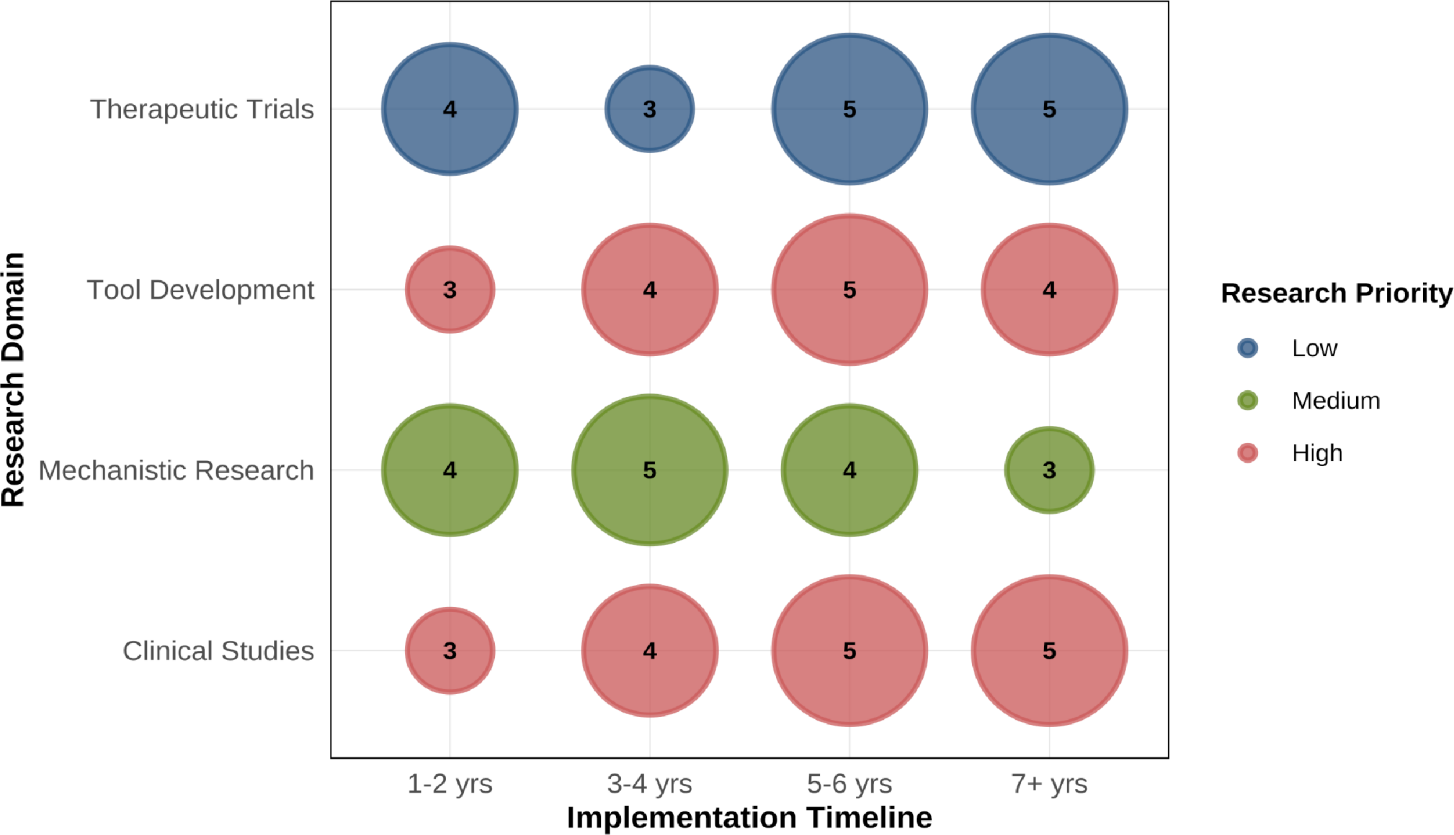
Strategic research roadmap for advancing the integration of obesity science with lung cancer immunotherapy. This bubble chart displays key research priorities for advancing the integration of obesity science with lung cancer immunotherapy. Research domains (y-axis) are plotted against implementation timelines (x-axis). Bubble size represents expected scientific impact (scale: 1-5). Bubble color indicates research priority (Low: blue; Medium: orange; High: green). High-priority initiatives include: large-scale prospective cohorts with serial body composition analysis, single-cell multi-omics profiling of adipose-tumor-immune interactions, artificial intelligence (AI)-powered integrative predictive models, precision dosing strategies based on pharmacokinetic/pharmacodynamic (PK/PD) modeling, and biomarker-enriched combination therapy trials targeting inflammation pathways (e.g., ICI plus anti-IL-6R antibodies).

Lastly, therapeutic optimization must diligently address personalized treatment strategy challenges for obese patients. The pharmacokinetics/pharmacodynamics aspect necessitates population pharmacokinetic model-based insight into how varying body compositions (high muscle, high fat, sarcopenic obesity) affect ICI drug distribution, metabolism, and clearance, providing scientific bases for dose adjustments among extremes of body weights (obesity or cachexia) (156), challenging current fixed-dose paradigms. Combination therapy strategies should prioritize biomarker-enriched umbrella or basket trial pursuits, for instance, tested ICI-anti IL-6 receptor antibody (like tocilizumab) in “high inflammation” phenotypic obese patients with high circulating IL-6 or VFA (157), assessing efficacy and safety; exploring whether concurrent normative nutrition support (e.g., high protein supplements) and individualized resistance training can reverse muscle loss, improve functional states, thus enhancing therapy tolerance and efficacy in patients with sarcopenia or sarcopenic obesity. Such explorations will lead us towards more finely tuned, dynamic lung cancer immune therapy eras underpinned by obesity’s physiopathological features guidance (158) (**Figures 6**, **7**).

## 7 Conclusion

Obesity, through its associated chronic low-grade systemic inflammation and immunometabolic disorder, deep and complexly engages the efficacy, toxicity, and biomarker networks of lung cancer immune checkpoint inhibitors (ICIs). It is not merely a background state but an active participant, significantly influencing ICI therapeutic efficacy by remodeling tumor microenvironments and altering systemic immune homeostasis, while increasing the risk of specific endocrine irAEs. Thoroughly understanding these multifaceted interactions is crucial for true precision medicine in the era of lung cancer immunotherapy.

Future research and practice must decisively transition from simple BMI correlations to deeper mechanism investigations and more precise multidimensional model constructions. Clinically, an integrated management model deeply blended with multidisciplinary expertise encompassing oncology, endocrinology, nutrition, rehabilitation, and pharmacy must be established for obese lung cancer patients, forming comprehensive, individualized management pathways. With an ever-deepening understanding of the obesity-immune axis interactions and the ongoing development of novel combination strategies and biomarkers, we expect to offer the growing cohort of obese lung cancer patients more personalized, safer, and effective immunotherapy regimens, ultimately improving this unique population’s long-term survival and quality of life.

## References

[1] Saiki M Takusagawa K Takahashi N Homma K Satoh T Furuya S . Effect of the combination of concomitant drugs on efficacy of immune checkpoint inhibitors in non-small cell lung cancer. Cancer Rep (Hoboken N.J.). (2025) 8:e70399. doi: 10.1002/cnr2.70399, PMID: 41195639 PMC12590243

[2] Shen Y Chen J Li XP . Research advances in immune checkpoint drugs for non-small cell lung cancer. J Drug Targeting. (2023) 31:700–13. doi: 10.1080/1061186X.2023.2235098, PMID: 37417910

[3] Hata T Yamada T Goto Y Amano A Negi Y Watanabe S . Regimen selection for chemoimmunotherapy in nonsquamous non-small cell lung cancer with low PD-L1 expression: A multicenter retrospective cohort study. Clin Lung Cancer. (2025) 26:e190–e198.e4. doi: 10.1016/j.cllc.2025.01.002, PMID: 39864962

[4] Vryza P Fischer T Mistakidi E Zaravinos A . Tumor mutation burden in the prognosis and response of lung cancer patients to immune-checkpoint inhibition therapies. Trans Oncol. (2023) 38:101788. doi: 10.1016/j.tranon.2023.101788, PMID: 37776617 PMC10542015

[5] Chen CY Chen WC Hung CM Wei YF . Chemotherapy or chemo-immunotherapy as first-line treatment for extensive-stage small-cell lung cancer: a meta-analysis. Immunotherapy. (2021) 13:1165–77. doi: 10.2217/imt-2021-0135, PMID: 34261336

[6] Ntikoudi E Kiagia M Boura P Syrigos KN . Hormones of adipose tissue and their biologic role in lung cancer. Cancer Treat Rev. (2014) 40:22–30. doi: 10.1016/j.ctrv.2013.06.005, PMID: 23870486

[7] Li F Gao Z . Obesity, chronic breast inflammation and carcinogenesis: Molecular pathways and clinical implications (Review). Int J Oncol. (2026) 68:12. doi: 10.3892/ijo.2025.5825, PMID: 41312718 PMC12674201

[8] Vita E Stefani A Piro G Mastrantoni L Cintoni M Cicchetti G . Leptin-mediated meta-inflammation may provide survival benefit in patients receiving maintenance immunotherapy for extensive-stage small cell lung cancer (ES-SCLC). Cancer Immunol Immunother: CII. (2023) 72:3803–12. doi: 10.1007/s00262-023-03533-0, PMID: 37668709 PMC10576666

[9] Davey MG Donlon NE Donnelly M Ryan EJ Ryan OK Reynolds IS . Evaluating the influence of the obesity paradox on survival outcomes in patients being treated surgically for rectal cancer-a systematic review and meta-analysis. Int J Colorect Dis. (2025) 40:180. doi: 10.1007/s00384-025-04957-z, PMID: 40826176 PMC12361308

[10] Wang F Zhou L Chen N Li X . The effect of pretreatment BMI on the prognosis and serum immune cells in advanced LSCC patients who received ICI therapy. Medicine. (2021) 100:e24664. doi: 10.1097/MD.0000000000024664, PMID: 33663076 PMC7909129

[11] Wang Z Yang M Zhao Y Wang J Tao M Yang J . Prognostic impact of body mass index in lung cancer patients receiving immune checkpoint inhibitors: an updated systematic review and meta-analysis. Technol Cancer Res Treat. (2025) 24:15330338251394573. doi: 10.1177/15330338251394573, PMID: 41236792 PMC12618815

[12] Jiang Y Li R Li X Zhang N . Risk factors of immune-mediated hepatotoxicity induced by immune checkpoint inhibitors in cancer patients: A systematic review and meta-analysis. Curr Oncol (Toronto Ont.). (2024) 31:7129–43. doi: 10.3390/curroncol31110525, PMID: 39590156 PMC11593173

[13] Chen L Chen H Chen Z Zhang K Zhang H Yi X . Visceral obesity, partly mediated by a reduction in HDL-C, increases the risk of asthma: Insights from NHANES and Mendelian randomization studies. Respir Med. (2025) 249:108482. doi: 10.1016/j.rmed.2025.108482, PMID: 41207538

[14] Wang P Zeng G Yan Y Zhang SY Dong Y Zhang Y . Disruption of adipocyte HIF-1α improves atherosclerosis through the inhibition of ceramide generation. Acta Pharm Sinica B. (2022) 12:1899–912. doi: 10.1016/j.apsb.2021.10.001, PMID: 35847503 PMC9279628

[15] Ahmadi-Kani Golzar F Fathi R Mahjoub S . High-fat diet leads to adiposity and adipose tissue inflammation: the effect of whey protein supplementation and aerobic exercise training. Appl Physiol Nutr Metab = Physiol Appl Nutr Et Metab. (2019) 44:255–62. doi: 10.1139/apnm-2018-0307, PMID: 30107135

[16] Sharma D Ravi RN Abdullah ADI Subramaniyan V . Therapeutic prospects of modulating TLR4/MAPK/ROS signalling in obesity-associated neuroinflammation. Biomed Pharmacother = Biomed Pharmacother. (2025) 193:118805. doi: 10.1016/j.biopha.2025.118805, PMID: 41314100

[17] LiuX LiX CaiD SunJ BaiW . Dietary patterns and testosterone balance: a review of clinical data and perspectives. J Adv Res. (2025) S2090-1232(25)00911-7. doi: 10.1016/j.jare.2025.11.016, PMID: 41274640

[18] Oh JM Yoon H Joo JY Im WT Chun S . Therapeutic potential of ginseng leaf extract in inhibiting mast cell-mediated allergic inflammation and atopic dermatitis-like skin inflammation in DNCB-treated mice. Front Pharmacol. (2024) 15:1403285. doi: 10.3389/fphar.2024.1403285, PMID: 38841363 PMC11150533

[19] Akhter N Madhoun A Arefanian H Wilson A Kochumon S Thomas R . Oxidative stress induces expression of the toll-like receptors (TLRs) 2 and 4 in the human peripheral blood mononuclear cells: implications for metabolic inflammation. Cell Physiol Biochem: Int J Exp Cell Physiol Biochem Pharmacol. (2019) 53:1–18. doi: 10.33594/000000117, PMID: 31162913

[20] Engin A . The pathogenesis of obesity-associated adipose tissue inflammation. Adv Exp Med Biol. (2017) 960:221–45. doi: 10.1007/978-3-319-48382-5_9, PMID: 28585201

[21] Frasca D Diaz A Romero M Thaller S Blomberg BB . Metabolic requirements of human pro-inflammatory B cells in aging and obesity. PloS One. (2019) 14:e0219545. doi: 10.1371/journal.pone.0219545, PMID: 31287846 PMC6615614

[22] Miyata Y Fukuhara A Otsuki M Shimomura I . Expression of activating transcription factor 2 in inflammatory macrophages in obese adipose tissue. Obes (Sil Spr Md.). (2013) 21:731–6. doi: 10.1002/oby.20274, PMID: 23712976

[23] Freemerman AJ Johnson AR Sacks GN Milner JJ Kirk EL Troester MA . Metabolic reprogramming of macrophages: glucose transporter 1 (GLUT1)-mediated glucose metabolism drives a proinflammatory phenotype. J Biol Chem. (2014) 289:7884–96. doi: 10.1074/jbc.M113.522037, PMID: 24492615 PMC3953299

[24] Dai J Zeng Z Wang L Yuan W Rocha KCE Park J . Obesity rewires CD8^+^T cell iron metabolism in adipose tissue to fuel metabolic inflammation. Metab: Clin Exp. (2025) 175:156438. doi: 10.1016/j.metabol.2025.156438, PMID: 41241023 PMC13573243

[25] Lee BC Lee J . Cellular and molecular players in adipose tissue inflammation in the development of obesity-induced insulin resistance. Biochim Biophys Acta. (2014) 1842:446–62. doi: 10.1016/j.bbadis.2013.05.017, PMID: 23707515 PMC3800253

[26] Engin A . Reappraisal of adipose tissue inflammation in obesity. Adv Exp Med Biol. (2024) 1460:297–327. doi: 10.1007/978-3-031-63657-8_10, PMID: 39287856

[27] Kang S Majid A Hammoudi N Lebeche D . Gene and drug-mediated SERCA2a activation restores cardiac function and metabolic balance in diabetic mice. Biomed Pharmacother = Biomed Pharmacother. (2025) 192:118617. doi: 10.1016/j.biopha.2025.118617, PMID: 41061581 PMC12919852

[28] Zhang BT Guo M Yang LR Zeng Y Jiang J . Mechanistic aspects of inflammation and oxidative stress and their association with thyroid cancer risk. Cancer Med. (2025) 14:e71030. doi: 10.1002/cam4.71030, PMID: 40620232 PMC12230506

[29] Fang X Wei J He X Lian J Han D An P . Quantitative association between body mass index and the risk of cancer: A global Meta-analysis of prospective cohort studies. Int J Cancer. (2018) 143:1595–603. doi: 10.1002/ijc.31553, PMID: 29696630

[30] Rodriguez-Monterrosas C Diaz-Aragon R Cortes-Reynosa P Salazar EP . Linoleic acid induces an increased response to insulin in MDA-MB-231 breast cancer cells. J Cell Biochem. (2018) 119:5413–25. doi: 10.1002/jcb.26694, PMID: 29363790

[31] Yang J Waldron RT Su HY Moro A Chang HH Eibl G . Insulin promotes proliferation and fibrosing responses in activated pancreatic stellate cells. Am J Physiol Gastrointest Liver Physiol. (2016) 311:G675–87. doi: 10.1152/ajpgi.00251.2016, PMID: 27609771 PMC5142202

[32] Bielawiec P Swierkot L Konstantynowicz-Nowicka K Chabowski A Błachnio-Zabielska A Harasim-Symbor E . Cannabigerol - A potent regulator of insulin sensitivity in rat’s skeletal muscle via targeting the sphingolipid metabolism and PI3K/Akt/mTOR pathway? Int J Biochem Cell Biol. (2025) 186:106819. doi: 10.1016/j.biocel.2025.106819, PMID: 40482851

[33] Oliveira KM Figueiredo LS Araujo TR Freitas IN Silva JN Boschero AC . Prolonged bisphenol-A exposure decreases endocrine pancreatic proliferation in response to obesogenic diet in ovariectomized mice. Steroids. (2020) 160:108658. doi: 10.1016/j.steroids.2020.108658, PMID: 32442623

[34] Wang J Wang JX Ruzemaimaiti S Wang YH Zhou Y Chen JX . Association of free fatty acids with long-term adverse outcomes in patients with premature myocardial infarction: A prospective cohort study. J Am Heart Assoc. (2025) 14:e042855. doi: 10.1161/JAHA.125.042855, PMID: 41128147 PMC12684601

[35] Dicks LMT . Key signals produced by gut microbiota associated with metabolic syndrome, cancer, cardiovascular diseases, and brain functions. Int J Mol Sci. (2025) 26:10539. doi: 10.3390/ijms262110539, PMID: 41226575 PMC12607389

[36] Cui Q Yang Q Wang Z Wan Y Jiang D Liao Y . GABA ameliorates diet-induced hepatic steatosis and insulin resistance by inhibiting macrophage activation. Diabet Obes Metab. (2025) 27:5085–98. doi: 10.1111/dom.16556, PMID: 40613220

[37] Sun S Wang R Song J Guan M Li N Zhang X . Blocking gp130 signaling suppresses autotaxin expression in adipocytes and improves insulin sensitivity in diet-induced obesity. J Lipid Res. (2017) 58:2102–13. doi: 10.1194/jlr.M075655, PMID: 28874440 PMC5665664

[38] Park H Kim M Kwon GT Lim DY Yu R Sung MK . A high-fat diet increases angiogenesis, solid tumor growth, and lung metastasis of CT26 colon cancer cells in obesity-resistant BALB/c mice. Mol Carcinog. (2012) 51:869–80. doi: 10.1002/mc.20856, PMID: 21919080

[39] Ray A Nkhata KJ Cleary MP . Effects of leptin on human breast cancer cell lines in relationship to estrogen receptor and HER2 status. Int J Oncol. (2007) 30:1499–509. doi: 10.3892/ijo.30.6.1499, PMID: 17487372

[40] Wang KX Shi DM Shi XL Wang JY Ai XH . Obesity promotes immunotherapy efficacy by up-regulating the glycolytic-mediated histone lactacylation modification of CD8^+^T cells. Front Pharmacol. (2025) 16:1533464. doi: 10.3389/fphar.2025.1533464, PMID: 40110127 PMC11920648

[41] Tokushige K Enzhi Y Tsuji-Yogo K Yu G Morimoto K Kuriyama K . Enhancement of suppressive function of Ly49^+^CD8^+^T cells in allogeneic immunity by CD80/86-CD28 blockade in mouse. J Heart Lung Transplant. (2025) 2498:02405–2. doi: 10.1016/j.healun.2025.11.017, PMID: 41297733

[42] Sessions DT Boulton DP Spoelstra NS Caino MC Yu M Goodspeed A . Androgen receptors promote oxidative phosphorylation and resistance to palmitate lipotoxicity in ER-mutant breast cancer. Endocrinology. (2025) 167:bqaf168. doi: 10.1210/endocr/bqaf168, PMID: 41216931 PMC12679916

[43] Ahmed F Khan S . Potential role of tumor-derived MIF in B-cell antigen presentation in lung adenocarcinoma: single-cell and TCGA analyses. Anticancer Res. (2025) 45:5369–87. doi: 10.21873/anticanres.17874, PMID: 41318120

[44] Zhang Z Lin Y Pan X Chen S . Inhibition of non-small cell lung cancer metastasis by knocking down APE1 through regulating myeloid-derived suppressor cells-induced immune disorders. Aging. (2024) 16:10435–45. doi: 10.18632/aging.205938, PMID: 38885059 PMC11236315

[45] Wang T Wang J Jiang H Ni M Zou Y Chen Y . Targeted regulation of tumor microenvironment through the inhibition of MDSCs by curcumin loaded self-assembled nano-filaments. Mater Today Bio. (2022) 15:100304. doi: 10.1016/j.mtbio.2022.100304, PMID: 35711288 PMC9194645

[46] Concato-Lopes VM Silva TF Detoni MB Cruz EMS Gonçalves MD da Silva Bortoleti BT . 3,3’,5,5’-Tetramethoxybiphenyl-4,4’diol triggers oxidative stress, metabolic changes, and apoptosis-like process by reducing the PI3K/AKT/NF-κB pathway in the NCI-H460 lung cancer cell line. Biomed Pharmacother = Biomed Pharmacother. (2024) 170:115979. doi: 10.1016/j.biopha.2023.115979, PMID: 38061138

[47] Zhou S Gu J Liu R Wei S Wang Q Shen H . Spermine alleviates acute liver injury by inhibiting liver-resident macrophage pro-inflammatory response through ATG5-dependent autophagy. Front Immunol. (2018) 9:948. doi: 10.3389/fimmu.2018.00948, PMID: 29770139 PMC5940752

[48] Tao Z McCall NS Wiedemann N Vuagniaux G Yuan Z Lu B . SMAC mimetic debio 1143 and ablative radiation therapy synergize to enhance antitumor immunity against lung cancer. Clin Cancer Res. (2019) 25:1113–24. doi: 10.1158/1078-0432.CCR-17-3852, PMID: 30352911

[49] Ogawa F Amano H Eshima K Ito Y Matsui Y Hosono K . Prostanoid induces premetastatic niche in regional lymph nodes. J Clin Invest. (2014) 124:4882–94. doi: 10.1172/JCI73530, PMID: 25271626 PMC4347225

[50] Yang F Wei Y Cai Z Yu L Jiang L Zhang C . Activated cytotoxic lymphocytes promote tumor progression by increasing the ability of 3LL tumor cells to mediate MDSC chemoattraction via Fas signaling. Cell Mol Immunol. (2015) 12:66–76. doi: 10.1038/cmi.2014.21, PMID: 24769795 PMC4654365

[51] Diehl B Kirchhoff A Hansmann F . CD86 is linked to apoptosis in canine histiocytic sarcoma. Front Vet Sci. (2025) 12:1546047. doi: 10.3389/fvets.2025.1546047, PMID: 40271486 PMC12014657

[52] Yang L Zhang Z Zhang Y Wang L Zheng S Li Y . Radiotherapy promotes M2 polarization of macrophages through the regulation of the PTEN/PI3K/AKT signaling pathway through miR-616-3p in lung cancer cell-derived exosomes. *In vitro* cellular & developmental biology. Animal. (2025) 61:1202–17. doi: 10.1007/s11626-025-01111-5, PMID: 41143935

[53] Zhu R Huang J Qian F . The role of tumor-associated macrophages in lung cancer. Front Immunol. (2025) 16:1556209. doi: 10.3389/fimmu.2025.1556209, PMID: 40079009 PMC11897577

[54] Zhao W Wang H Zhang X Zhang L Pu W Ma Y . Effects of IFN-γ on the immunological microenvironment and TAM polarity in stage IA non-small cell lung cancer and its mechanisms. BMC Pulmon Med. (2024) 24:46. doi: 10.1186/s12890-023-02809-6, PMID: 38254043 PMC10802021

[55] Park SH . Ethyl Acetate Fraction of Adenophora triphylla var. japonica Inhibits Migration of Lewis Lung Carcinoma Cells by Suppressing Macrophage Polarization toward an M2 Phenotype. J Pharmacopunct. (2019) 22:253–9. doi: 10.3831/KPI.2019.22.034, PMID: 31970023 PMC6970570

[56] Wang C Fan X Fang S Tan F Zhang Y Duan X . Expression and clinical significance of MMP-1 and MMP-7 in pneumonia, pulmonary fibrosis, and lung cancer. J Visualiz Exp: JoVE. (2025) 10.3791/69107. doi: 10.3791/69107, PMID: 41212837

[57] Lou J Xiang Z Zhu X Li J Jin G Cui S . Gut microbiota constituents may affect hypertrophic scarring risk through interaction with specific immune cells in a two-step, two-sample Mendelian randomization study. Sci Rep. (2025) 15:20656. doi: 10.1038/s41598-025-07455-y, PMID: 40596527 PMC12216485

[58] Lou J Xiang Z Zhu X Fan Y Li J Jin G . A two-step, two-sample Mendelian randomization analysis investigating the interplay between gut microbiota, immune cells, and melanoma skin cancer. Medicine. (2024) 103:e40432. doi: 10.1097/MD.0000000000040432, PMID: 39533622 PMC11557063

[59] Lou J Cui S Li J Jin G Fan Y Huang N . Causal relationship between the gut microbiome and basal cell carcinoma, melanoma skin cancer, ease of skin tanning: evidence from three two-sample mendelian randomisation studies. Front Immunol. (2024) 15:1279680. doi: 10.3389/fimmu.2024.1279680, PMID: 38304424 PMC10830803

[60] Sun E Meng X Kang Z Gu H Li M Tan X . Zengshengping improves lung cancer by regulating the intestinal barrier and intestinal microbiota. Front Pharmacol. (2023) 14:1123819. doi: 10.3389/fphar.2023.1123819, PMID: 36992837 PMC10040556

[61] Zhao Y Wang B Wei X Liu D Wang R Xie S . Impact of gut microbiota dysbiosis on intestinal barrier integrity and systemic inflammation in a pre-eclampsia mouse model. Microb Pathog. (2025) 209:108053. doi: 10.1016/j.micpath.2025.108053, PMID: 40975229

[62] Tian R Ding Y Zhang S Li M Wang Y Wu Q . Chlorogenic acid alleviates the intestinal barrier dysfunction and intestinal microbiota disorder induced by cisplatin. Front Microbiol. (2025) 16:1508891. doi: 10.3389/fmicb.2025.1508891, PMID: 40104593 PMC11919278

[63] Li Q Chen L Wang R . Exercise reshapes gut microbiota to ameliorate core symptoms in PCOS: molecular mechanisms and therapeutic implications. Front Endocrinol. (2025) 16:1652731. doi: 10.3389/fendo.2025.1652731, PMID: 41234235 PMC12605003

[64] Han B Chai Q Chen Q Liu M Wang T Zhang Y-L . Sodium butyrate inhibits colorectal cancer development by reducing M2 macrophage polarization and PD-L1 expression. mSystems. (2025) 10:e0069225. doi: 10.1128/msystems.00692-25, PMID: 41251471 PMC12710303

[65] Li S Duan Y Luo S Zhou F Wu Q Lu Z . Short-chain fatty acids and cancer. Trends Cancer. (2025) 11:154–68. doi: 10.1016/j.trecan.2024.11.003, PMID: 39638744

[66] Arpaia N Campbell C Fan X Dikiy S Van Der Veeken J deRoos P . Metabolites produced by commensal bacteria promote peripheral regulatory T-cell generation. Nature. (2013) 504:451–5. doi: 10.1038/nature12726, PMID: 24226773 PMC3869884

[67] Mao C Fan W Liu J Yang F Li W Li L . Targeting HDAC and PARP enhances STING-dependent antitumor immunity in STING-deficient tumor. Adv Sci (Weinheim Baden-Wurttemberg Germany). (2025) 12:e07904. doi: 10.1002/advs.202507904, PMID: 40789065 PMC12591105

[68] Luu M Weigand K Wedi F Breidenbend C Leister H Pautz S . Regulation of the effector function of CD8^+^T cells by gut microbiota-derived metabolite butyrate. Sci Rep. (2018) 8:14430. doi: 10.1038/s41598-018-32860-x, PMID: 30258117 PMC6158259

[69] Xu Y Qian T Pan H Gu Y Xu M Bai C . Akkermansia muciniphila-derived outer membrane protein Amuc_1100 promotes acetylation of RING Finger Protein 181 promoter and mediates Autophagy Related 7 ubiquitination to activate CD8^+^T cells in lung adenocarcinoma. Int J Biol Macromol. (2025) 330:147773. doi: 10.1016/j.ijbiomac.2025.147773, PMID: 40975344

[70] Lou J Zhu X Xiang Z Fan Y Song J Huang N . The efficacy and safety of negative pressure wound therapy in paediatric burns: a systematic review and meta-analysis of randomized controlled trials. BMC pediatrics, (2024) 24:807. doi: 10.1186/s12887-024-05302-z, PMID: 39696096 PMC11653751

[71] Nam JW Kim SE Lee KM Jung YW Oh JM Lee WR . Association between body-mass index change and lung cancer risk in Korea: nested case-control study. BMC Cancer. (2025) 25:1805. doi: 10.1186/s12885-025-15122-8, PMID: 41286693 PMC12642259

[72] Fidelle M Chen H Montégut L Boulate D Abdayem P Martineau M . Increased plasma concentrations of acyl-coenzyme A binding protein (ACBP) predict future lung cancer development in smokers at risk of cardiovascular disease. Mol Cancer. (2025) 24:296. doi: 10.1186/s12943-025-02507-3, PMID: 41291789 PMC12645689

[73] Kichenadasse G Miners JO Mangoni AA Rowland A Hopkins AM Sorich MJ . Association between body mass index and overall survival with immune checkpoint inhibitor therapy for advanced non-small cell lung cancer. JAMA Oncol. (2020) 6:512–8. doi: 10.1001/jamaoncol.2019.5241, PMID: 31876896 PMC6990855

[74] Fang J Li Q Xu N Yang X Zhang Q Chen Y . Systemic inflammation biomarkers can identify high tumor mutation burden in lung adenocarcinoma. BMC Cancer. (2025) 25:1543. doi: 10.1186/s12885-025-14894-3, PMID: 41068630 PMC12512448

[75] Su L Li X Yang Q Wang Q Gong Y Yang W . Association between advanced lung cancer inflammation index and postoperative mortality in lung cancer patients: a retrospective cohort study. Eur J Med Res. (2025) 30:e13820. doi: 10.1186/s40001-025-03694-x, PMID: 41408364 PMC12822094

[76] Krejčí D Šubrt A Opálka P Krejčí J Šibalová M Svatoň M . Impact of baseline body mass index on immunotherapy outcomes in patients with non-small-cell lung cancer. Anticancer Res. (2025) 45:5579–86. doi: 10.21873/anticanres.17892, PMID: 41318117

[77] Han R Li J Wang Y He T Zheng J He Y . Low BMI patients with advanced EGFR mutation-positive NSCLC can get a better outcome from metformin plus EGFR-TKI as first-line therapy: A secondary analysis of a phase 2 randomized clinical trial. Chin Med J Pulmon Crit Care Med. (2023) 1:119–24. doi: 10.1016/j.pccm.2023.04.006, PMID: 39170825 PMC11332817

[78] Ihara Y Sawa K Imai T Bito T Shimomura Y Kawai R . Immunotherapy and overall survival among patients with advanced non-small cell lung cancer and obesity. JAMA Netw Open. (2024) 7:e2425363. doi: 10.1001/jamanetworkopen.2024.25363, PMID: 39093562 PMC11297387

[79] Sikkema BJ Baart SJ Paats MS Smit EF Schols AMWJ Mathijssen RHJ . Body weight gain associated with alectinib in patients with ALK+ Non-small cell lung cancer: pooled analysis of individual patient data from four prospective clinical trials. J Clin Oncol. (2025) 43:641–50. doi: 10.1200/JCO-24-01579, PMID: 39661917

[80] Kono K Tanino R Tsubata Y Haque EF Isobe T Okimoto T . Microtubule inhibitors induce cross-resistance to osimertinib through caMKII activation in EGFR-mutated NSCLC. Cancer Sci. (2025) 10.1111/cas.70274. doi: 10.1111/cas.70274, PMID: 41316890 PMC12861095

[81] Yamada M Warabi E Oishi H Lira VA Okutsu M . Muscle-derived IL-1β regulates EcSOD expression via the NBR1-p62-Nrf2 pathway in muscle during cancer cachexia. J Physiol. (2024) 602:4215–35. doi: 10.1113/JP286460, PMID: 39167700

[82] Ikenoue S Tamai J Akita K Otani T Kasuga Y Tanaka M . Origins of obesity in the womb: Fetal adiposity and its determinants. J Obstet Gynaecol Res. (2024) 50:2178–82. doi: 10.1111/jog.16114, PMID: 39385507 PMC11608846

[83] Zhang H Huang J Li Y Jin W Wei J Ma N . Celastrol-loaded ginsenoside Rg3 liposomes boost immunotherapy by remodeling obesity-related immunosuppressive tumor microenvironment in melanoma. Acta Pharm Sinica B. (2025) 15:2687–702. doi: 10.1016/j.apsb.2025.03.017, PMID: 40487651 PMC12145075

[84] O’Sullivan D van der Windt GJ Huang SC Curtis JD Chang CH Buck MD . Memory CD8(+) T cells use cell-intrinsic lipolysis to support the metabolic programming necessary for development. Immunity. (2014) 41:75–88. doi: 10.1016/j.immuni.2014.06.005, PMID: 25001241 PMC4120664

[85] Khemka A Clasen SC Loehrer PJ Roberts AR Golzarri-Arroyo L Badve SS . Cardiovascular disease in thymic cancer patients. Front Cardiovasc Med. (2024) 11:1393631. doi: 10.3389/fcvm.2024.1393631, PMID: 39346095 PMC11427757

[86] Safarova Yantsen Y Nessipbekova A Syzdykova A Olzhayev F Umbayev B Kassenova A . Strontium- and copper-doped ceramic granules in bone regeneration-associated cellular processes. J Funct Biomater. (2024) 15:352. doi: 10.3390/jfb15110352, PMID: 39590555 PMC11595051

[87] Hua T Wu Q Huang Z Cai J . Fufang zhenzhu tiaozhi capsule alleviates hypothalamic endoplasmic reticulum stress and leptin resistance through autophagy modulation in DIO rats. J Ethnopharmacol. (2025) 351:120056. doi: 10.1016/j.jep.2025.120056, PMID: 40436122

[88] Lyu X Liu J Liu Z Wu Y Zhu P Liu C . Anti-inflammatory effects of reticuline on the JAK2/STAT3/SOCS3 and p38 MAPK/NF-κB signaling pathway in a mouse model of obesity-associated asthma. Clin Respir J. (2024) 18:e13729. doi: 10.1111/crj.13729, PMID: 38286741 PMC10799233

[89] Shin JH Shin SH . A comprehensive review of naringenin, a promising phytochemical with therapeutic potential. J Microbiol Biotechnol. (2024) 34:2425–38. doi: 10.4014/jmb.2410.10006, PMID: 39572023 PMC11733549

[90] Emond C DeVito MJ Diliberto JJ Birnbaum LS . The influence of obesity on the pharmacokinetics of dioxin in mice: an assessment using classical and PBPK modeling. Toxicol Sci. (2018) 164:218–28. doi: 10.1093/toxsci/kfy078, PMID: 29596651 PMC6016688

[91] Kopetz S Boni V Kato K Raghav KPS Vieito M Pallis A . Precemtabart tocentecan, an anti-CEACAM5 antibody-drug conjugate, in metastatic colorectal cancer: a phase 1 trial. Nat Med. (2025) 31:3504–13. doi: 10.1038/s41591-025-03843-z, PMID: 40739424 PMC12532702

[92] Mohanty SS Mohanty PK . Obesity as potential breast cancer risk factor for postmenopausal women. Genes Dis. (2019) 8:117–23. doi: 10.1016/j.gendis.2019.09.006, PMID: 33997158 PMC8099684

[93] Takenaka Y Oya R Takemoto N Inohara H . Predictive impact of sarcopenia in solid cancers treated with immune checkpoint inhibitors: a meta-analysis. J Cachex Sarcopenia Muscle. (2021) 12:1122–35. doi: 10.1002/jcsm.12755, PMID: 34337889 PMC8517360

[94] Bezel P Valaperti A Steiner U Scholtze D Wieser S Vonow-Eisenring M . Evaluation of cytokines in the tumor microenvironment of lung cancer using bronchoalveolar lavage fluid analysis. Cancer Immunol Immunother: CII. (2021) 70:1867–76. doi: 10.1007/s00262-020-02798-z, PMID: 33394095 PMC8195789

[95] Daoudlarian D Segot A Latifyan S Bartolini R Joo V Mederos N . Tocilizumab and immune signatures for targeted management of cytokine release syndrome in immune checkpoint therapy. Ann Oncol. (2025) 36:444–59. doi: 10.1016/j.annonc.2024.12.004, PMID: 39701282

[96] Kuusisalo S Iivanainen S Koivunen JP . Association of anti-PD-(L)1 treatment duration to efficacy in advanced solid tumors: a single center retrospective study. Ann Med. (2025) 57:2476729. doi: 10.1080/07853890.2025.2476729, PMID: 40091413 PMC11915729

[97] Minami S Ihara S Tanaka T Komuta K . Sarcopenia and visceral adiposity did not affect efficacy of immune-checkpoint inhibitor monotherapy for pretreated patients with advanced non-small cell lung cancer. World J Oncol. (2020) 11:9–22. doi: 10.14740/wjon1225, PMID: 32095185 PMC7011908

[98] Khan AW Chandra S Mahadevia H Subramanian J Ponvilawan B Bansal D . Immune checkpoint inhibitor-induced hemophagocytic lymphohistiocytosis in lung cancer: a case series. Explor Target Anti-Tumor Ther. (2025) 6:1002347. doi: 10.37349/etat.2025.1002347, PMID: 41244945 PMC12615973

[99] Park JE Jo J Youk J Kim M Yoon SH Keam B . Prognostic utility of body composition parameters based on computed tomography analysis of advanced non-small cell lung cancer treated with immune checkpoint inhibitors. Insights Into Imaging. (2023) 14:182. doi: 10.1186/s13244-023-01532-4, PMID: 37880430 PMC10600077

[100] Takahara Y Abe R Sumito N Tanaka T Ishige Y Shionoya I . Investigation of response factors for monotherapy with immune checkpoint inhibitors in non-small cell lung cancer patients with PD-L1 expression <50. Thorac Cancer. (2023) 14:2754–60. doi: 10.1111/1759-7714.15059, PMID: 37536667 PMC10518233

[101] Cortellini A Bersanelli M Santini D Buti S Tiseo M Cannita K . Another side of the association between body mass index (BMI) and clinical outcomes of cancer patients receiving programmed cell death protein-1 (PD-1)/Programmed cell death-ligand 1 (PD-L1) checkpoint inhibitors: A multicentre analysis of immune-related adverse events. Eur J Cancer. (2020) 128:17–26. doi: 10.1016/j.ejca.2019.12.031, PMID: 32109847

[102] Huang Y Soon YY Aminkeng F Tay SH Ang Y Kee ACL . Risk factors for immune-related adverse events from anti-PD-1 or anti-PD-L1 treatment in an Asian cohort of nonsmall cell lung cancer patients. Int J Cancer. (2022) 150:636–44. doi: 10.1002/ijc.33822, PMID: 34562273

[103] Zhang H Zheng J Ren C Ye C Wu X Lv X . Risk factors of immune-related endocrine toxicities in non-small cell lung cancer patients treated with pembrolizumab and its impact on patient outcomes: a multicenter retrospective study. BMC Pulmon Med. (2025) 25:111. doi: 10.1186/s12890-025-03570-8, PMID: 40082871 PMC11905628

[104] Durá-Travé T Gallinas-Victoriano F . Hyper-androgenemia and obesity in early-pubertal girls. J Endocrinol Invest. (2022) 45:1577–85. doi: 10.1007/s40618-022-01797-4, PMID: 35412268 PMC9270300

[105] Akhter N CarlLee T Syed MM Odle AK Cozart MA Haney AC . Selective deletion of leptin receptors in gonadotropes reveals activin and GnRH-binding sites as leptin targets in support of fertility. Endocrinology. (2014) 155:4027–42. doi: 10.1210/en.2014-1132, PMID: 25057790 PMC4164926

[106] Rossi S Silvetti F Bordoni M Ciarloni A Salvio G Balercia G . Pembrolizumab-induced thyroiditis, hypophysitis and adrenalitis: A case of triple endocrine dysfunction. JCEM Case Rep. (2024) 2:luae200. doi: 10.1210/jcemcr/luae200, PMID: 39498471 PMC11532647

[107] Westerbacka J Yki-Järvinen H Vehkavaara S Häkkinen AM Andrew R Wake DJ . Body fat distribution and cortisol metabolism in healthy men: enhanced 5beta-reductase and lower cortisol/cortisone metabolite ratios in men with fatty liver. J Clin Endocrinol Metab. (2003) 88:4924–31. doi: 10.1210/jc.2003-030596, PMID: 14557475

[108] Xiong J Li J Wang Z Lu S Liang S Xiao W . Case report: pembrolizumab-induced acute type 1 diabetes mellitus and diabetic ketoacidosis in a perioperative esophageal squamous cell carcinoma patient. AME Case Rep. (2025) 9:61. doi: 10.21037/acr-24-159, PMID: 40330922 PMC12053383

[109] Tang Y Zhao Z Wang X Zuo W Zhang B Yuan T . A case of pembrolizumab-induced fulminant Type 1 diabetes mellitus in breast cancer. Immunotherapy. (2021) 13:483–9. doi: 10.2217/imt-2020-0222, PMID: 33626915

[110] Maaloul I Aloulou H Bessghaier W Ameur SB Chabchoub I Khalfallah R . Primary adrenal insufficiency in children excluding congenital adrenal hyperplasia: insights from 33-year single-center experience in Tunisia. Arch Pediatr. (2025) S0929-693X(25)00019-3. doi: 10.1016/j.arcped.2024.10.010, PMID: 39875217

[111] Yuan Y Cui S Hou Y Meng X Huang Y Xu F . Associations between sex hormones and obesity-related indicators: results from the NHANES and Mendelian randomization study. Eur J Med Res. (2025) 30:1216. doi: 10.1186/s40001-025-03470-x, PMID: 41310874 PMC12683816

[112] Connolly AM Schierbecker J Renna R Florence J . High dose weekly oral prednisone improves strength in boys with Duchenne muscular dystrophy. Neuromuscul Disord: NMD. (2002) 12:917–25. doi: 10.1016/s0960-8966(02)00180-3, PMID: 12467746

[113] Elzouki AY Jaiswal OP . Long-term, small dose prednisone therapy in frequently relapsing nephrotic syndrome of childhood. Effect on remission, statural growth, obesity, and infection rate. Clin Pediatr. (1988) 27:387–92. doi: 10.1177/000992288802700807, PMID: 3135974

[114] Liu C Li X . Role of leptin and adiponectin in immune response and inflammation. Int Immunopharmacol. (2025) 161:115082. doi: 10.1016/j.intimp.2025.115082, PMID: 40516255

[115] Metz CN Brines M Xue X Chatterjee PK Adelson RP Roth J . Increased plasma lipopolysaccharide-binding protein and altered inflammatory mediators reveal a pro-inflammatory state in overweight women. BMC Women’s Health. (2025) 25:57. doi: 10.1186/s12905-025-03588-4, PMID: 39930423 PMC11809003

[116] Durmus A Kesici U Genc MS Mazlum AF Ercan LD Duman MG . Can hemogram parameters be used as a biomarker for thyroid carcinomas? Ann Ital Di Chir. (2025) 96:1571–7. doi: 10.62713/aic.4054, PMID: 41243539

[117] Tekin S Basmaci N Lay İ Gürlek A . Anthropometric and metabolic assessment in adults with Down syndrome: the need for novel indices and tailored criteria. Turk J Med Sci. (2025) 55:1161–73. doi: 10.55730/1300-0144.6071, PMID: 41234441 PMC12611377

[118] Yildiz LM Kızılkaya B Cüre O . Comprehensive evaluation of non-standard inflammatory and metabolic indices in obesity: A single-center retrospective study. Healthc (Basel Switzerland). (2025) 13:2946. doi: 10.3390/healthcare13222946, PMID: 41302334 PMC12652752

[119] Corsini EM Wang Q Tran HT Mitchell KG Antonoff MB Hofstetter WL . Peripheral cytokines are not influenced by the type of surgical approach for non-small cell lung cancer by four weeks postoperatively. Lung Cancer (Amsterdam Netherlands). (2020) 146:303–9. doi: 10.1016/j.lungcan.2020.06.022, PMID: 32619781

[120] Wang C Zheng B Lin C Li X Li R Zhao L . Effects of liposomal bupivacaine for erector spinae plane block on perioperative immune function and analgesia in thoracoscopic lung cancer surgery. Am J Cancer Res. (2025) 15:3728–39. doi: 10.62347/SQHE7607, PMID: 40948529 PMC12432565

[121] Recio-Boiles A Galeas JN Goldwasser B Sanchez K Man LMW Gentzler RD . Enhancing evaluation of sarcopenia in patients with non-small cell lung cancer (NSCLC) by assessing skeletal muscle index (SMI) at the first lumbar (L1) level on routine chest computed tomography (CT). Support Care Cancer. (2018) 26:2353–9. doi: 10.1007/s00520-018-4051-2, PMID: 29417293 PMC5984123

[122] Turcott JG Miyagui SM Gutiérrez Torres S Cárdenas-Fernández D Caballé-Perez E Rios-Garcia E . Sarcopenia as a predictive factor for carboplatin toxicity in patients with advanced non-small cell lung cancer. Nutr Cancer. (2024) 76:985–93. doi: 10.1080/01635581.2024.2382390, PMID: 39066469

[123] Völter F Wehlte L Resuli B Walter J Daisenberger L Ingenerf M . Elevated FDG uptake in non-tumorous lung regions does not predict immune checkpoint inhibitor-related pneumonitis in lung cancer patients. Front Oncol. (2025) 15:1563030. doi: 10.3389/fonc.2025.1563030, PMID: 40909944 PMC12405330

[124] Spielvogel CP Lazarevic A Zisser L Haberl D Eseroglou C Beer L . Artificial intelligence-enabled opportunistic identification of immune checkpoint inhibitor-related adverse events using [18F]FDG PET/CT. Eur J Nucl Med Mol Imaging. (2025) 52:4963–71. doi: 10.1007/s00259-025-07364-2, PMID: 40439712 PMC12589324

[125] Zheng L Bian Y Hu Y Tian C Zhang X Li S . Baseline 18F-FDG PET/CT parameters in predicting the efficacy of immunotherapy in non-small cell lung cancer. Front Med. (2025) 12:1477275. doi: 10.3389/fmed.2025.1477275, PMID: 39958820 PMC11825783

[126] Chen Z Chen X Ju L Li Y Li W Pang H . Establishing a predictive model for tumor mutation burden status based on 18F-FDG PET/CT and clinical features of non-small cell lung cancer patients. Trans Lung Cancer Res. (2024) 13:2269–81. doi: 10.21037/tlcr-24-416, PMID: 39430315 PMC11484715

[127] Lou J Cui S Huang N Jin G Chen C Fan Y . Efficacy of probiotics or synbiotics in critically ill patients: A systematic review and meta-analysis. Clin Nutr ESPEN. (2024) 59:48–62. doi: 10.1016/j.clnesp.2023.11.003, PMID: 38220407

[128] Flück CE Kariyawasam D Ceppi F Shalitin S Busiah K . Endocrine-related adverse conditions in pediatric patients treated with immune checkpoint inhibitors: A position statement from the clinical practice committee of the european society for pediatric endocrinology. Horm Res Paediatr. (2025) 1–4. doi: 10.1159/000546146, PMID: 40300586 PMC12176352

[129] Gao Y Qi F Zhou W Jiang P Hu M Wang Y . Liquid biopsy using plasma proteomics in predicting efficacy and tolerance of PD-1/PD-L1 blockades in NSCLC: a prospective exploratory study. Mol Biomed. (2025) 6:51. doi: 10.1186/s43556-025-00291-6, PMID: 40659985 PMC12260144

[130] Orillard E Adhikari A Malouf RS Calais F Marchal C Westeel V . Immune checkpoint inhibitors plus platinum-based chemotherapy compared to platinum-based chemotherapy with or without bevacizumab for first-line treatment of older people with advanced non-small cell lung cancer. Cochrane Database Syst Rev. (2024) 8:CD015495. doi: 10.1002/14651858.CD015495, PMID: 39136258 PMC11320659

[131] Qin B Xin L Liang C Li L Song Q Long Y . Efficacy and safety of anti-PD-1 inhibitor versus anti-PD-L1 inhibitor in first-line treatment of extensive-stage small cell lung cancer: a multicenter retrospective study. BMC Cancer. (2024) 24:100. doi: 10.1186/s12885-024-11833-6, PMID: 38233798 PMC10795417

[132] Al Heyasat A Chaudhry MS Alkharabsheh M Bani Amer M Poojary I . Pembrolizumab-induced hypophysitis: A rare immune-related adverse event in a patient with metastatic non-small cell lung cancer. Cureus. (2025) 17:e82701. doi: 10.7759/cureus.82701, PMID: 40400852 PMC12094803

[133] Li X Cui W Duan C Zhang C . Causal relationship between metabolic traits and risk of NSCLC: A two-sample mendelian randomization analysis. J Cancer. (2025) 16:4139–46. doi: 10.7150/jca.109913, PMID: 41210690 PMC12595243

[134] Harre P Hohenberger K Krammer S Yang Z Tausche P Willar J . Dietary ω-3 polyunsaturated fatty acids (PUFAs) reduce cholesterol-driven non-small cell lung cancer (NSCLC) progression in mouse models of disease. Commun Med. (2025) 5:432. doi: 10.1038/s43856-025-01193-y, PMID: 41131338 PMC12549838

[135] McKenzie J Sneath E Trinh A Nolan M Spain L . Updates in the pathogenesis and management of immune-related enterocolitis, hepatitis and cardiovascular toxicities. Immuno-Oncol Technol. (2024) 21:100704. doi: 10.1016/j.iotech.2024.100704, PMID: 38357008 PMC10865026

[136] Verheijden RJ Cabané Ballester A Smit KC van Eijs MJM Bruijnen CP van Lindert ASR . Physical activity and checkpoint inhibition: association with toxicity and survival. J Natl Cancer Inst. (2024) 116:573–9. doi: 10.1093/jnci/djad245, PMID: 38001030 PMC10995850

[137] Dimitriou F Staeger R Ak M Maissen M Kudura K Barysch MJ . Frequency, treatment and outcome of immune-related toxicities in patients with immune-checkpoint inhibitors for advanced melanoma: results from an institutional database analysis. Cancers. (2021) 13:2931. doi: 10.3390/cancers13122931, PMID: 34208218 PMC8230729

[138] Presti M Westergaard MCW Draghi A Chamberlain CA Gokuldass A Svane IM . The effects of targeted immune-regulatory strategies on tumor-specific T-cell responses *in vitro*. Cancer Immunol Immunother: CII. (2021) 70:1771–6. doi: 10.1007/s00262-020-02760-z, PMID: 33165629 PMC10992602

[139] Stroud CR Hegde A Cherry C Naqash AR Sharma N Addepalli S . Tocilizumab for the management of immune mediated adverse events secondary to PD-1 blockade. J Oncol Pharm Pract. (2019) 25:551–7. doi: 10.1177/1078155217745144, PMID: 29207939

[140] Færch K Blond MB Bruhn L Amadid H Vistisen D Clemmensen KKB . The effects of dapagliflozin, metformin or exercise on glycaemic variability in overweight or obese individuals with prediabetes (the PRE-D Trial): a multi-arm, randomised, controlled trial. Diabetologia. (2021) 64:42–55. doi: 10.1007/s00125-020-05306-1, PMID: 33064182

[141] Villani LA Smith BK Marcinko K Ford RJ Broadfield LA Green AE . The diabetes medication Canagliflozin reduces cancer cell proliferation by inhibiting mitochondrial complex-I supported respiration. Mol Metab. (2016) 5:1048–56. doi: 10.1016/j.molmet.2016.08.014, PMID: 27689018 PMC5034684

[142] Lou J Xiang Z Zhu X Li J Jin G Cui S . Skin microbiota and diabetic foot ulcers. Front Microbiol. (2025) 16:1575081. doi: 10.3389/fmicb.2025.1575081, PMID: 40666801 PMC12261678

[143] Panja S Mantri P Johnson KE Andrade-Martinez JS Yang SR Deshpande A . Passenger mutations link cellular origin and transcriptional identity in human lung adenocarcinomas. Nat Genet. (2025) 57:3066–74. doi: 10.1038/s41588-025-02418-5, PMID: 41310231 PMC13425454

[144] Skoulidis F Borghaei H Garon EB Leal TA Kaufman J Liu SV . Rationale and design for a phase IIIb trial of first-line tremelimumab plus durvalumab versus pembrolizumab, in combination with chemotherapy, in patients with non-squamous metastatic non-small-cell lung cancer and mutations or co-mutations in STK11, KEAP1, or KRAS: the TRITON study. Ther Adv Med Oncol. (2025) 17:17588359251386611. doi: 10.1177/17588359251386611, PMID: 41209621 PMC12592654

[145] Lee JM . Neoadjuvant immunotherapy. Thorac Surg Clinics. (2026) 36:1–8. doi: 10.1016/j.thorsurg.2025.08.003, PMID: 41260723

[146] Luo T Li H Chen A Ouyang T Wu M Yang M . A population-based nomogram for prognostic assessment in advanced lung cancer following progression with immune checkpoint inhibitor. J Thorac Dis. (2025) 17:5078–94. doi: 10.21037/jtd-2025-165, PMID: 40809275 PMC12340266

[147] Yanai M Sakamoto T Nonaka T Nakada T Matsuoka S Moriyama S . Pseudo-hyperprogression of Malignant pleural mesothelioma treated with nivolumab. Internal Med (Tokyo Japan). (2025) 64:2386–9. doi: 10.2169/internalmedicine.4807-24, PMID: 39924236 PMC12393948

[148] Ding H Yuan M Yang Y Xu XS . Longitudinal genomic profiling using liquid biopsies in metastatic nonsquamous NSCLC following first line immunotherapy. NPJ Precis Oncol. (2025) 9:5. doi: 10.1038/s41698-024-00797-2, PMID: 39779891 PMC11711381

[149] Gao Q Wang Z Liu C Zhang H Fan L Fan J . Integration of spatial and single-cell transcriptomic analysis uncovers cellular and molecular alterations in the hypertensive brain. Life Sci. (2025) 385:124107. doi: 10.1016/j.lfs.2025.124107, PMID: 41297664

[150] Mao C . Sphingolipid metabolism dysregulation: A cause for lung cancer development, progression, and resistance to therapies. Chin Med J Pulmon Crit Care Med. (2025) 3:88–96. doi: 10.1016/j.pccm.2025.05.002, PMID: 40677415 PMC12266267

[151] Zhang W Liao Y Lou J Zhuang M Yan H Li Q . CircRNA_Maml2 promotes the proliferation and migration of intestinal epithelial cells after severe burns by regulating the miR-93-3p/FZD7/Wnt/β-catenin pathway. Burns Trauma. (2022) 10:tkac009. doi: 10.1093/burnst/tkac009, PMID: 35265724 PMC8900685

[152] Khan S Upadhyay S Kauser S Hasan GM Lu W Waters M . Redefining the diagnostic and therapeutic landscape of non-small cell lung cancer in the era of precision medicine. J Clin Med. (2025) 14:8021. doi: 10.3390/jcm14228021, PMID: 41303058 PMC12653151

[153] Zhao Q Hu L Ji H . Advancing small cell lung cancer metastasis research: innovations in preclinical mouse models. Cancer Metast Rev. (2025) 44:86. doi: 10.1007/s10555-025-10301-2, PMID: 41318700

[154] Liu L Yang L Li H Shang T Liu L . The tumor microenvironment in lung cancer: Heterogeneity, therapeutic resistance and emerging treatment strategies (Review). Int J Oncol. (2026) 68:11. doi: 10.3892/ijo.2025.5824, PMID: 41312736 PMC12674202

[155] Zhao Y Song X Luo W Xie F Shen J He J . Post-translational modifications of protein and lung cancer. Front Oncol. (2025) 15:1667200. doi: 10.3389/fonc.2025.1667200, PMID: 41306527 PMC12643890

[156] Zarić M Čanović P Živković Zarić R Protrka S Glišić M . The three musketeers in cancer therapy: pharmacokinetics, pharmacodynamics and personalised approach. J Pers Med. (2025) 15:516. doi: 10.3390/jpm15110516, PMID: 41295218 PMC12653432

[157] Shenoy PV Baburaj G Damerla RR Pai A Mailankody S Munisamy M . Influence of genetic polymorphisms on gefitinib pharmacokinetics and adverse drug reactions in non-small cell lung cancer patients. Cancer Metast Rev. (2025) 44:82. doi: 10.1007/s10555-025-10299-7, PMID: 41199076 PMC12592279

[158] Kumbhare M Pagere ND Ide B Gode H Shaikh A . Targeting ROS1 in NSCLC: clinical advances and future directions of taletrectinib. Zhongguo Ying Yong Sheng Li Xue Za Zhi = Zhongguo Yingyong Shenglixue Zazhi = Chin J Of Appl Physiol. (2025) 41:e20250025. doi: 10.62958/j.cjap.2025.025, PMID: 41178326

